# One-pot synthesis of carboxymethyl-dextran coated iron oxide nanoparticles (CION) for preclinical fMRI and MRA applications

**DOI:** 10.1016/j.neuroimage.2021.118213

**Published:** 2021-06-09

**Authors:** Manasmita Das, Esteban A. Oyarzabal, Lars Chen, Sung-Ho Lee, Neal Shah, Gabby Gerlach, Weiting Zhang, Tzu-Hao Harry Chao, Nathalie Van Den Berge, Carolyn Liu, Carrie Donley, Stephanie A. Montgomery, Yen-Yu Ian Shih

**Affiliations:** a Center for Animal MRI, University of North Carolina, Center for Animal MRI, 125 Mason Farm Road, CB# 7513, Chapel Hill, NC 27599, USA; b Biomedical Research Imaging Center, University of North Carolina, Chapel Hill, NC, USA; c Department of Neurology, University of North Carolina, Chapel Hill, NC, USA; d Curriculum in Neurobiology, University of North Carolina, Chapel Hill, NC, USA; e Center for Nanotechnology in Drug Delivery, University of North Carolina, Chapel Hill, NC, USA; f Department of Pathology and Laboratory Medicine, University of North Carolina, Chapel Hill, NC, USA; g Medical Image and Signal Processing Group, Ghent University, Ghent, Belgium

**Keywords:** CION, Intravascular, Iron oxide, MRI contrast agent, fMRI, CBV, Angiography

## Abstract

Superparamagnetic iron-oxide nanoparticles are robust contrast agents for magnetic resonance imaging (MRI) used for sensitive structural and functional mapping of the cerebral blood volume (CBV) when administered intravenously. To date, many CBV-MRI studies are conducted with Feraheme, manufactured for the clinical treatment of iron-deficiency. Unfortunately, Feraheme is currently not available outside the United States due to commercial and regulatory constraints, making CBV-MRI methods either inaccessible or very costly to achieve. To address this barrier, we developed a simple, one-pot recipe to synthesize Carboxymethyl-dextran coated Iron Oxide Nanoparticles, namely, “CION”, suitable for preclinical CBV-MRI applications. Here we disseminate a step-by-step instruction of our one-pot synthesis protocol, which allows CION to be produced in laboratories with minimal cost. We also characterized different CION-conjugations by manipulating polymer to metal stoichiometric ratio in terms of their size, surface chemistry, and chemical composition, and shifts in MR relaxivity and pharmacokinetics. We performed several proof-of-concept experiments *in vivo*, demonstrating the utility of CION for functional and structural MRI applications, including hypercapnic CO_2_ challenge, visual stimulation, targeted optogenetic stimulation, and microangiography. We also present evidence that CION can serve as a cross-modality research platform by showing concurrent *in vivo* optical and MRI measurement of CBV using fluorescent-labeled CION. The simplicity and cost-effectiveness of our one-pot synthesis method should allow researchers to reproduce CION and tailor the relaxivity and pharmacokinetics according to their imaging needs. It is our hope that this work makes CBV-MRI more openly available and affordable for a variety of research applications.

## Introduction

Blood-oxygenation-level-dependent functional MRI (BOLD-fMRI) is among the most commonly used noninvasive imaging techniques for functional brain mapping (Bandettini et al., 1992; Kim and Ogawa, 2012; Kwong et al., 1992; Logothetis, 2008; Ogawa et al., 1990, 1992; Uğurbil, 2012). This technique indirectly measures neuronal activity by detecting associated changes in regional blood oxygenation. In most cases, regional cerebral blood flow (CBF) increases adjacent to neuronal activity, resulting in a regional reduction of paramagnetic deoxyhemoglobin and subsequent enhancement of the BOLD-fMRI signal. Though BOLD-fMRI has been widely utilized, this technique suffers from low specificity and sensitivity. In preclinical animal models, cerebral blood volume (CBV)-weighted fMRI enhanced by exogenous contrast agents is a more sensitive alternative to BOLD (Kim et al., 2013; Lu et al., 2004b; Mandeville, 2012; Zhao et al., 2006). Additionally, CBV represents direct physiological changes to vascular tone, making this metric more easily interpretable. CBV-fMRI has been used for robust functional neuronal circuit mapping in preclinical animal models (Albaugh et al., 2016; Decot et al., 2017; Giorgi et al., 2017; He et al., 2018; Kim et al., 2013; Silva et al., 2007; Van Den Berge et al., 2017; Zhao et al., 2015, 2012), surgical planning (Cha, 2009; Nguyen et al., 2017), preoperative brain mapping in neurosurgical patients (Hart et al., 2016), clinical outcome assessment (Nasseri et al., 2014; Varallyay et al., 2018) and preclinical development of new therapeutic agents for various brain diseases (Artem Shatillo et al., 2020; Borsook et al., 2006; Carmichael et al., 2018). As this technique is capable of normalizing relaxivity changes to derive percent CBV changes, the resulting values can be independent of magnetic field strength and echo time, permitting comparisons across various acquisition settings (Fukuda et al., 2006; Harel et al., 2006; Jin and Kim, 2008b; Kim et al., 2013; Lu et al., 2004a, 2004c; Mandeville et al., 1998; Shih et al., 2014; Wu et al., 2004).

The first-ever CBV-fMRI study in human was conducted using serial injections of gadolinium chelate (Belliveau et al., 1991). Although this study demonstrated a significant task-induced vasodilation in primary visual cortex and set the foundation for MR-based functional brain mapping, the rapid washout of contrast required repeated dosing and thus lacked the much needed “steady-state” feature for efficient measurement. To date, most clinically-approved gadolinium-based contrast agents (GDCA) possess relatively short circulation half-life, which limits their use for fMRI applications. In addition, GDCA have been associated with long-term toxicity in the kidneys and brain (Gulani et al., 2017; Kuo et al., 2007; Malayeri et al., 2016; Wei et al., 2017). Development of MRI contrast agents based on superparmagnetic iron-oxide nanoparticles (SPIO, mean hydrodynamic diameter > 50 nm) or ultrasmall superparamagnetic iron-oxide nanoparticles (USPIO, mean hydrodynamic diameter ≤ 50 nm) have been increasingly used for diagnostic and theranostic purposes (Atanasijevic and Jasanoff, 2007; Atanasijevic et al., 2006; Chen et al., 2014, 2015; Himmelreich et al., 2005; Jung and Jacobs, 1995; Jung et al., 2014; Senpan et al., 2009; Shapiro et al., 2005, 2004; Stark et al., 1988; Wei et al., 2017; Weissleder et al., 1990a, 1990b; Wu et al., 2004). Careful manipulation of synthesis variables such as the size, magnetization and surface properties of SPIO/USPIO can be easily tailored to achieve longer intravascular half-life, higher MR sensitivity and better biocompatibility, as compared to commercially available GDCA. Typically composed of polymer-coated magnetite or maghemite nanocrystals, SPIO and USPIO-based nanoprobes can produce sensitive negative contrast via T_2_ and T_2_* shortening that are usually stronger than GDCA (Atanasijevic et al., 2006; Bulte and Kraitchman, 2004). With GDCA and standard measurement conditions, 10–100 μM concentrations are typically required for robust visualization in MRI, whereas SPIO/USPIO based contrast agents are detectable at sub-micromolar concentrations (Atanasijevic et al., 2006). Unfortunately, except for Resovist® (FUJIFILM RI Farma Co., Ltd., Kyobashi, Tokyo, Japan) used for liver imaging in very few countries such as Japan, currently no FDA-approved iron-oxide contrast agent is available for clinical MRI (Daldrup-Link, 2017). Numerous first generation USPIO contrast agents such as Ferumoxtran-10 (Combidex®/Sinerem®), Feruglose (Clariscan™) or Supravist™ were withdrawn from the market following unfavorable safety profiles in phase II or III clinical trials (Wang, 2011).

Recently, most CBV-MRI studies were conducted with Ferumoxytol (Feraheme™), a second generation USPIO manufactured by AMAG pharmaceuticals, Inc. This formulation is comprised of an aqueous colloidal suspension of USPIO, coated with polyglucose sorbitol Carboxymethyl ether (PSC, a Carboxymethylated and reduced dextran composed of 20–22 glucose units) and made isotonic with mannitol (Bullivant et al., 2013). Although Feraheme has been clinically accepted for the parenteral therapy of iron-deficiency anemia in adult patients with chronic kidney diseases (Allkemper et al., 2002; Lu et al., 2010; Schiller et al., 2011), the FDA has not yet approved its use for MRI. Nevertheless, the long blood pool residence time of Feraheme has made it a preferred contrast agent for CBV mapping in a variety of preclinical models including rodents (Idée et al., 2006; Jung et al., 2014; Lu et al., 2005; Mueggler et al., 2001; Sieber et al., 2008; Weinstein et al., 2010, 2000; Weissleder et al., 1990b; Zhao et al., 2001), rabbits (Berry et al., 1996; D’Arceuil et al., 1999; Xia et al., 2014), cats (Zhang et al., 2010; Zhao et al., 2007, 2005) and nonhuman primates (Mandeville et al., 2001; Oliveira et al., 2016; Prince et al., 1995; Schroeter et al., 2014). Feraheme has only been used in investigational ‘off-label’ high resolution magnetic resonance angiography (Hope et al., 2015; Stoumpos et al., 2018), resting state fMRI (D’Arceuil et al., 2013) and steady-state CBV mapping (Christen et al., 2013; Varallyay et al., 2013, 2018) in humans. Routine application of Feraheme in clinical and even preclinical settings is challenging due to numerous reasons. Firstly, to ensure patient safety, many research and clinical institutes as well as regulatory agencies in different countries may require an investigational new drug application to be approved for any ‘off-label’ application (Narang et al., 2013; Shi et al., 2013). Secondly, post marketing surveillance data has demonstrated the risk of serious and fatal hypersensitivity reactions including anaphylaxis in patients receiving Feraheme (Food and Administration, 2015; McCormack, 2012). In 2010, Takeda Pharmaceuticals was granted the right to develop and commercialize Ferumoxytol outside the US and its territories including Switzerland and the European Union (EU) under the trade name Rienso. Unfortunately, in 2015, the marketing authorization for Rienso was withdrawn in Europe at the mutual request of Takeda and AMAG pharmaceuticals due to commercial and regulatory reasons (Bullivant et al., 2013).

The sudden unavailability of Feraheme outside the US led to the suspension of many CBV-MRI studies in the field. Although some preclinical iron-oxide based nanoformulations are available in the market that could be used for CBV imaging, these formulations are relatively costly, especially considering the high doses required for *in vivo* MRI studies (15–30 mg/kg) (Albaugh et al., 2016; Decot et al., 2017; Giorgi et al., 2017; He et al., 2018; Kim et al., 2013; Silva et al., 2007; Van Den Berge et al., 2017; Zhao et al., 2015, 2012). Synthesizing a blood pool agent that simultaneously exhibit long shelf-life, high T_2_ relaxivity and prolonged intravascular retention has proven challenging since *in vivo* efficacy and safety of nanoparticulate contrast agents depend on various parameters including size, magnetization, nature of surface functionality and polymer coverage on the surface of nanoparticles as well as their pharmacokinetics and biodistribution following intravenous administration (Arami et al., 2015; Hoshyar et al., 2016; Wang et al., 2015). In this study, we report a simple, one-pot recipe for the synthesis of **C**arboxymethyl-dextran coated **I**ron **O**xide **Na**noparticles, termed “**CION**”, for reliable, preclinical MRI applications. We demonstrate methods to (1) tailor the material and pharmacokinetic properties of CION according to specific imaging requirements; (2) validate their suitability for various *in vivo* applications including high resolution micro-magnetic resonance angiography (μMRA) and stimulus-evoked CBV-fMRI; (3) evaluate their toxicity; and (4) conjugate CION with a fluorescent dye for cross-modality imaging. We also compared and validated our results against Feraheme. At the time of preparation of this manuscript, the cost of all equipment needed to perform in-house CION synthesis was ~ $3000 USD and the supplies/reagents needed to synthesize a batch of 150 ml of 20 mg Fe/ml CION is ~ $330 USD. The ease of production and cost-effectiveness of this CION synthesis protocol should make CBV-MRI platform openly accessible and affordable to preclinical MRI research laboratories.

## Materials and methods

### Synthesis of CION

#### Methods.

**Fig. 1.**
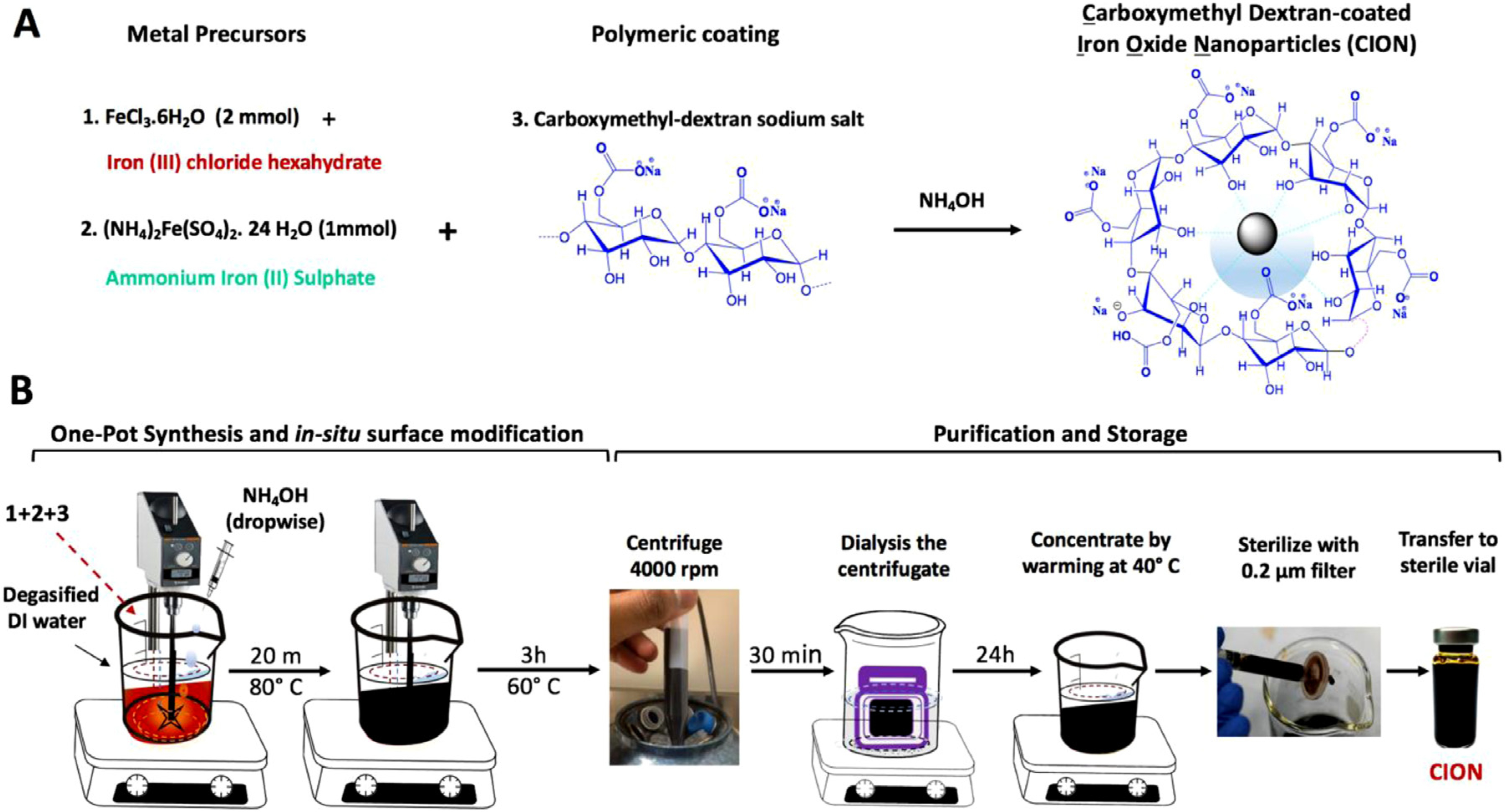
Synthesis of CION. **(A)** An illustration chemical conjugates used for CION synthesis. **(B)** A schematic methodology depicting the one-pot synthesis of CION via *in-situ*, alkali-mediated co-precipitation of metal precursors in presence of carboxymethyl dextran (CMD) sodium salt.

We synthesized CION using controlled, alkali-mediated co-precipitation of iron (III) chloride hexahydrate (FeCl_3_•6H_2_O) and ammonium iron (II) sulfate [FeSO_4_. (NH_4_)_2_SO_4_.24 H_2_O] in presence of Carboxymethyl-dextran (CMD) Sodium Salt (average molecular weight 10–20 kDa) (Fig. 1). All equipment, reagents/chemicals and laboratory supplies required for CION synthesis including their corresponding sources and tentative cost have been listed in Table S1. In order to determine the effect of reaction variables on the physicochemical and pharmacokinetic (PK) properties of CION, we initially prepared several formulations with varied stoichiometric ratio of CMD and iron-precursors. These compositions are designated as CION_120000_, CION_60000_, CION_30000_, CION_17143_ and CION_12000,_ where the numbers in subscript represents the theoretical molar ratio of total iron precursor to polymer used for the synthesis, considering the molecular weight of CMD to be 10 kDa. Among these, we selected three compositions to illustrate how the MR relaxivity and PK properties of CION are adjustable by changing reaction variables. Our step-by-step synthesis are described as follows, illustrated in Fig. 1, and further detailed in Supplementary Video 1.

1. Dissolve FeCl_3_. 6H_2_O (4 mmol) and (NH_4_)_2_Fe(SO_4_)_2_•6H_2_O (2 mmol) in 20 ml of de-ionized water deoxygenated via thermo-mechanical degasification prior to addition of the reagents. Stir the resulting solution at 800–1000 rpm and 80–90°C in a 250 ml beaker.
2. To synthesize CION_60000_, CION_17143_ and CION_12000_, dissolve 1, 3.5 and 5 g of CMD, respectively in minimum volume of de-ionized water. Add the resultant solution slowly to the mixture of ferric and ferrous salt solution.
3. Initially, an orange precipitate would be observed, which would dissolve following the addition of 30% aqueous ammonia. Keep adding ammonia solution until all the precipitate dissolve and a silky, black colloid is obtained.
4. Add a Phosphate Buffered Saline (PBS) tablet to the resultant colloid for tonicity adjustment and stir the mixture for additional 3 h at 80°C to complete the nucleation and growth of nanoparticles.
5. Thereafter, cool the reaction mixture and centrifuge at 4000 rpm for 30 min in-order to facilitate separation of unreacted precursors and/or bigger particles, if any.
6. Transfer the nanoparticle solution to a Slide-A-Lyzer™ dialysis cassette/flask (Molecular weight cut-off: 20 kDa) and dialyze against PBS. Replace the dialysis media with fresh PBS every 2 h. After 24 h, transfer the colloidal suspension to a beaker and concentrate by evaporation on a hot plate at 40°C.
7. Adjust the final volume to approximately 20 mg Fe/ml theoretical concentration. Caution: Give special attention to the reaction mixture as soon as the volume reaches close to 15 mg Fe/ml to avoid gelling.
8. Sterilize the resultant colloid by passing through 0.2 μm PTFE syringe filters.
9. Transfer the final sterile solution was to a sterile vial.
10. Store the final formulation at room temperature.

### Validation of CION

#### Physicochemical Characterization.

To demonstrate the physicochemical properties of CION resulting from the above-mentioned one-pot synthesis protocol, we provide characterization data using procedures detailed below.

#### Iron concentration.

Iron (Fe) concentration per milliliter (ml) for every synthesized batch of CION were determined using inductively coupled plasma mass spectrometry (ICP-MS) with a Nexion 300D ICP-MS equipped with collision cell and autosampler. Samples for ICP-MS analysis were directly submitted to the Nanomedicines Characterization Core Facility at the University of North Carolina (UNC) at Chapel Hill. To determine iron concentration per unit volume of the colloidal sample, a given volume of CION (50–100 μl) was digested with a given volume of concentrated nitric acid. Thereafter, a small portion of this digested sample was transferred to de-ionized water. ICP-MS data was acquired after further diluting this sample with DI water. Iron concentration in the analyte was determined by running a calibration curve with a known concentration of Fe. The intensity of the ^57^Fe isotope was considered for quantification of Fe concentration. ICP-MS data was reported as concentration in ppb, which was converted into mg/ml and multiplied with total dilution factor to determine the amount of Fe present per unit volume of the contrast agent.

#### Size and morphology.

The size and morphology of nanoparticles were examined using a JEOL 2010F FAsTEM, a high-resolution transmission electron microscope (HRTEM) equipped with a 2KX2K Gatan CCD bottom mount camera and electron dispersive X-ray (EDX) attachment. The TEM samples were prepared by depositing a few drops of the respective nanoparticle preparations ultrasonically dispersed in water for 2 min on separate carbon-coated copper grids and air-dried at room temperature. An acceleration voltage of 200 kV and variable magnification (20kX–500kX) were used for imaging the samples. The hydrodynamic diameter (z-average) and polydispersity index (PDI) of the formulations were determined by dynamic light scattering (DLS) using a Malvern Zetasizer (Malvern Instruments, Malvern, UK). Hydrodynamic diameter measurements were performed in clear, disposable zeta cells, after diluting 1 μl of CION with 999 μl of DI water. Each sample was analyzed three times for a total period of analysis of 10 min. Samples with visible aggregation were not subjected to TEM or DLS analysis. Only nanoparticle preparations that were stable in physiologically compatible buffers at high concentrations (> 5 mg Fe/ml) for at least a week were characterized.

#### Surface chemistry and composition.

The surface chemistry of CION was studied using attenuated total reflection Fourier transform infrared (ATR-FTIR) spectroscopy using a Thermo Nicolet Nexux FTIR model 870 spectrometer. Measurements were done with colloidal samples. The surface composition of different CION preparations were obtained by X-ray photoelectron spectroscopy (XPS) using Al K_*α*_ excitation source in a Kratos Axis Ultra DLD X-ray Photoelectron Spectrometer with a base pressure of *ca*. 6 × 10^−9^ torr. Pass energies of 80 eV and 20 eV were used for survey and high-resolution scans, respectively. Atomic concentrations were determined in the Kratos Vision software. Shirley backgrounds were used and the software calculated the area under each peak. Relative sensitivity factors were used to determine atomic concentrations using the Kratos Vision software.

### MRI

#### Equipment.

All MRI studies were conducted using a Bruker 9.4 T/30-cm bore small animal MRI system. *In vitro* relaxometry studies were performed with a homemade sample holder and a quadrature transceiver volume coil with 35 mm inner-diameter. For *in vivo* rat MRI studies, animals were affixed on a homemade, custom-built holder and headpiece equipped with ear and tooth bars to minimize motion artifacts during imaging. A homemade surface coil (internal diameter ~1.6 cm) was placed directly over the head of the subject and used as a transceiver. Mouse imaging was performed on a commercial mouse holder using a 72 mm quad-transmit only volume coil and a quad-receive only mouse brain coil.

#### Animals.

All *in vivo* MRI studies and surgical procedures were conducted in accordance with the Guide for the Care and Use of Laboratory Animals, as adopted by the National Institutes of Health, and approved by UNC IACUC. Pharmacokinetic profile of different CION formulations as well as the suitability of CION_17143_ for CBV-fMRI and multimodal applications were assessed on adult Sprague Dawley (SD) rats between 400 and 500 g weight (*n* = 20). The efficacy of CION_17143_ as a μMRA contrast agent and its toxicity evaluation compared to Feraheme were tested on adult C57BL/6 J mice (*n* = 11) between 26 and 32 g of body weight.

#### In vitro Relaxometry.

Relaxometry of CION_60000_, CION_17143_ and CION_12000_ and Feraheme were evaluated *in vitro* with a custom-designed sample holder (Fig. 3A) securing the samples in the volume coil. The samples tested were serially diluted and placed into a 1 ml syringe. From each test sample, we prepared 7 different Fe concentrations: 0 (saline), 3.48746E-06, 6.97491E-06, 1.74373E-05, 3.48746E-05, 6.97491E-05 and 0.000174373 M. Images of these phantoms were acquired using a standard spin-echo sequence featuring multiple repetition time (TR: 5000, 3000, 1500, 800, 400 and 200 ms) and echo time (TE: 11, 33, 55, 77 and 99 ms) with acquisition matrix of 256×256, field of view (FOV) of 40 mm^2^, slice thickness of 1 mm, and 3 averages.

#### Pharmacokinetic MRI.

For pharmacokinetic (PK) assessment, each rat was anesthetized with urethane (1.3 g/kg) prior to experimentation. Throughout the experiment, heart rate and oxygen saturation (SpO_2_) were continuously monitored using a non-invasive physiological monitoring system equipped with MR-compatible sensors and maintained within normal ranges (300–350 bpm and above 96%, respectively). Warm-water circulating pads were used to maintain rectal temperatures at 37±0.5 °C. After initial positioning and pre-scan adjustments, magnetic field homogeneity was optimized using Bruker FASTMAP shimming up to second order with an isotropic 7 mm^3^ voxel encompassing the imaging slices. A fast-spin-echo T_2_-weighted image was taken in the mid-sagittal plane to localize the anatomical position by identifying the anterior commissure at 0.36 mm posterior to bregma. For PK assessment, we used a single shot gradient-echo (GE) echo-planar-imaging (EPI) sequence with acquisition matrix =80 × 80, FOV= 2.56 × 2.56 cm, slice thickness= 1 mm, spectral bandwidth = 250 kHz, TR = 1000 ms, TE = 8.3 ms, flip angle = 52°. To ensure consistency of the imaging location, 8 coronal slices were acquired with the fourth anterior slice aligned with the anterior commissure. To monitor the clearance of CION from cerebrovasculature, EPI data was continuously acquired for 260 min and animals were intravenously injected with CION_60000_ (*n* = 2), CION_17143_ (*n* = 5), CION_12000_ (*n* = 3) and Feraheme (*n* = 3) at a dose of 30 mg Fe/kg through a tail vein catheter at 20 min after scan onset.

#### In vivo fMRI applications.

Among all the CION formulations, only CION_17143_ presented optimal physicochemical and pharmacokinetic properties for long-term vascular imaging applications (See *Results and Discussions* for details). To further verify if CION_17143_ offers robust contrast for dynamic CBV-fMRI study, we performed several fMRI experiments including CO_2_ challenge, visual stimulation, and optogenetic stimulation in adult SD rats. These studies employed well-established procedures with well-documented biological outcomes (Chan et al., 2017; 2012; Keilholz et al., 2006; Leong et al., 2016; Liang et al., 2015; Lin et al., 2008, 2009; Schmid et al., 2017, 2016; Wu et al., 2003; Yu et al., 2016; Zhao et al., 2005, 2006), and were not designed to address any specific biological or neural circuit related questions. Instead, we focused on demonstrating the utilization of CION for these experiments (*n* = 1 or 2 for each study). Animal preparations were similar to those recently published (Albaugh et al., 2016; Broadwater et al., 2018; Kao et al., 2014; Van Den Berge et al., 2017). Briefly, each rat was endotracheally intubated and ventilated with 1–1.5% isoflurane in medical air using a small animal ventilator (CWE Inc., SAR-830/PA, Armore, PA). Ventilation rate and volume were adjusted as required to maintain EtCO_2_ within a range of 2.6–3.2% and SpO_2_ above 96%. All other experimental protocols including animal monitoring, physiology maintenance, imaging hardware and pulse sequences were described in the PK measurement section. During all fMRI experiments, a cocktail of dexmedetomidine (0.1 mg/kg/h) and pancuronium bromide (1.0 mg/kg/hr) were infused intraperitoneally to the animals. The level of isoflurane anesthesia was lowered to 0.5% at 30 min after the start of infusion (Fukuda et al., 2013). All CBV-weighted fMRI data were acquired by the EPI protocol described in the Pharmacokinetic MRI section, with a bolus dose of CION_17143_ (20 mg Fe/kg, i.v.) via a tail vein catheter. Specifically, these experiments include:

- **Hypercapnia-evoked fMRI with CION**_**17143**_. To evaluate the effect of hypercapnia on steady-state CBV, animals were exposed to 5% CO_2_ (in 95% air) challenge using a 180 s OFF, 180 s ON, and 360 s OFF paradigm post-CION injection.
- **Visual stimulation-evoked fMRI with CION**_**17143**_. To evaluate the effect of visual stimulation, animals were exposed to 10 Hz blue light visual stimuli using a 40 s OFF, 10 s ON, and 40 s OFF paradigm repeating 8 times in a post-CION injection scan.
- **Optogenetic fMRI with CION**_**17143**_. To stimulate pyramidal neurons in the motor cortex (M1), rats were microinjected with 1 μl of purified and concentrated adeno-associated virus (~10^12^ infections units per ml, packaged by the UNC Vector Core Facility) encoding channelrhodopsin-2 (ChR2) under Ca^2+^/calmodulin-dependent protein kinase II promoter (CaMKII*α*) and an enhanced yellow fluorescent protein (EYFP), or sham vector controls that do not express ChR2 (*n* = 2 each). Injection coordinate from bregma were anterior/posterior (AP): +3 mm, medial/lateral (ML): ±2.5 mm, and dorsal/ventral (DV): −1.5 mm. A control vector was injected into the M1 of the contralateral hemisphere. A 26-gauge injection needle connected to a 2 μl Hamilton syringe was used; each microinjection was performed over 10 min followed by another 10 min to allow diffusion of viral particles away from the injection site. After virus injection, chronic optical fibers were stereotactically implanted directly over the site of virus injection (DV: −1.0 mm) for light delivery. The time from virus injection to the start of the MRI experiments was 4–5 weeks. Before sending the animals inside the magnet, optical fibers were connected via 3 m patch cables to a solid-state laser (473 nm wavelength) located outside of the magnet room delivering ~10 mW light to virus-transfected region. We acquired stimulus-evoked fMRI data for 100 s during which light stimulation was applied in a 20 s OFF, 10 s ON, 30 s OFF, 10 s ON, and 30 s OFF paradigm. All subjects underwent 2–5 repeated trials at 40 Hz and 5 ms pulse width.

#### High resolution μMRA.

High resolution microangiography was performed in a C57Bl/6 J wild-type mouse anesthetized with 1.3 g/kg bolus of urethane (injected i.p. 1 h prior to imaging). We used a 3D Fast-Low-Angle-SHot (FLASH) sequence with acquisition matrix = 384×384×156, FOV = 19.2 × 19.2 × 7.8 mm, spectral bandwidth = 50 kHz, TR = 50 ms, TE = 5.9 ms, flip angle = 13°. This sequence allows mapping cerebrovasculature with an isotropic spatial resolution of 50 μm^3^. Two separate acquisition (each with four repetitions) were acquired before and after CION_17143_ injection (30 mg Fe/kg, i.v.), respectively.

#### Cross-modality application.

To demonstrate the utility of CION platform for cross-modality application, we conjugated CION with a red-fluorescent dye, Rhodamine B (Laser Grade, +99%, ACROS Organics™). Rhodamine B has been used for CBV measurement with two-photon microscopy (Tomita et al., 2011; Verant et al., 2007). Rhodamine B is usually conjugated with the antibiofouling polymer Dextran to prevent rapid bloodstream clearance. In this study, we conjugated Rhodamine B with CION with instead of Dextran, which not only endowed additional stability to the dye for optical measurements of local CBV with high temporal resolution (> 10 Hz), but also enabled brain-wide CBV mapping using simultaneous fMRI. Additionally, Rhodamine B can be measured simultaneously with a genetically encoded green fluorescent calcium indicator GCaMP6f—facilitating direct assessment of neuronal activity.

- **Animal Preparation and Surgery:** We expressed GCaMP6f in neurons of the forepaw area of the somatosensory cortex (coordinate from bregma: AP = +1.0, ML = +3.7, DV = −1.2 mm) in rats (*n* = 4) via an intracranial injection of genetically engineered Adeno-Associated Viruses (AAV9-Syn-GCaMP6f-WPRE-SV40, #100,837-AAV9, Addgene, Cambridge, MA). Rats were anesthetized initially by 5% isoflurane and maintained by a constant flow of 2–3% isoflurane mixed with medical air. Rectal temperature was continuously monitored and maintained within 37 ± 0.5 °C using a feedback-controlled heating pad. Rats were head-fixed to a stereotactic frame (Kopf Instruments, Model 962, Tujunga, CA). An incision was made to the exposed skull surface and a burr hole were prepared at skull above the infusion coordinate. Microinjection was performed at a flow rate of 0.1 *μ*l/min, and an additional 10 min was given for virus diffusion prior to slow retraction of the micro syringe needle. Subsequently, the incision was closed with 4.0 silk suture, and the rats were recovered from anesthesia. After two weeks of incubation, an optical fiber (#CFMLC12U-20, Thorlabs, Newton, NJ) was chronically implanted at a position that was at 0.3 mm above the virus injection site. Four MR-compatible miniature brass screws (Item #94070A031, McMaster Carr, Atlanta, GA) were anchored to the skull. Thereafter, the surface of the skull was covered with dental cement to seal implanted components and the wound was sutured to further protect the surgical site. Lidocaine jelly (#1,043,377, Henry Schein, Queens, NY) was applied around the surgical wound to relieve pain and prevent the rat from scratching the wound. Meloxicam (1.5 mg/kg for 2 days; #049,755, Henry Schein, Queens, N) was also given for further pain relief. Lincomycin hydrochloride (30 mg/kg, i.m.) was administrated to prevent infection. Rats were allowed at least 1 week for recovery before any further experiment.
- **Setting up the fiber-photometry system for simultaneous fMRI and optical imaging.** We used an established fiber-photometry setting (Meng et al., 2018) for concurrent fMRI applications. Briefly, we used a 488 nm continuous wave (CW) laser (OBIS 488LS-60, Coherent, Santa Clara, CA) to excite GCaMP6f, and a 561 nm CW laser (OBIS 561LS-60, Coherent, Inc.) to excite Rhodamine B. These two lasers were aligned, combined, and subsequently launched into a fluorescence cube (DFM1, Thorlabs, Newton, NJ). The fluorescence cube contained a dichroic mirror (ZT488/561rpc, Chroma Technology Corp) to reflect and launch the combined laser beam into the core of a multi-mode optical fiber patch cable (#M43L02, Thorlabs, Newton, NJ), which was connected to the chronically implanted optical fiber probe of rats. The emission fluorescence collected from the same fiber probe travelled back along the patch cable into the fluorescence cube, passed through the dichroic mirror and an emission filter (ZET488/561 m, Chroma Technology Corp, Bellows Falls, VT), and launched into the core of a multi-mode patch cable (M200L02S-A, Thorlabs, Newton, NJ) connected to a spectrometer (QE Pro-FL, Ocean Optics, Largo, FL). Spectral data was sampled at 10 Hz and processed using a UI software from Ocean View (Ocean Optics, Largo, FL). When trigger mode was setup in Ocean View, the spectrometer recording was synchronized with MRI TTL via an Arduino micro-controller board.
- **Simultaneous fMRI and fiber photometry in GCaMP6f rats.** The animal preparation for CBV-fMRI experiment was the same as aforementioned. Briefly, a pair of electrodes was inserted into the left forepaw between 2nd and 3rd / 3rd and 4th digits for electric stimulation. The stimulation paradigm was 60 s OFF - 30 s ON - 60 s OFF - 30 s ON - 60 s OFF. During the on blocks, electric stimulation was applied using a constant current stimulation isolator (#A365RC, WPI, Sarasota, FL) with following parameters: 3 mA, 9 Hz, 0.5 ms pulse width. The stimulation isolator was synchronized with MRI using a National Instruments DAQ board to receive TTL triggers from MRI scanner, and send out stimulus triggers according to the stimulus paradigm set in a homemade program.

### MRI data processing and analysis

#### Relaxometry.

*In vitro* relaxometric analyses were performed using a custom-written MATLAB (Math-Works, Natick, MA) script. Signal intensity corresponding to each Fe concentration at different TR and TE was extracted by placing identical size of region of interests (ROIs) on each phantom image. Relaxation curves for T_1_ calculation was obtained by plotting signal intensities (SI) against variable TRs corresponding to the shortest TE. The T_1_ recovery was plotted using a one phase exponential association fitting: SI = a*(1-exp (−b*TR)) + *c* where a, b, c denotes the fitting parameters. Similarly, the T_2_ values were obtained by plotting SI against variable TEs corresponding to the longest TR. The T_2_ decay was plotted using a one phase exponential decay: SI = a*exp(−b*TE). The relaxivity values (r_1_= (1/ΔT_1_)/[Fe] and r_2_ = (1/ΔT_2_)/[Fe]) were determined by linear fitting to the relaxation rates (1/ΔT_1_ and 1/ΔT_2_) *vs* Fe concentration [Fe].

#### Pharmacokinetics and Clearance.

All EPI data acquired for pharmacokinetic evaluation of CION were corrected for motion using Analysis of Functional Neuroimages (AFNI) software. Because the clearance of CION is a slow process with respect to our EPI temporal resolution, the time-series EPI data were smoothed by moving average using a 30 s sliding window to avoid noise influence on pharmacokinetic assessment. The resultant images were motion corrected, skull-stripped, and co-registered to a template space using custom-written Matlab scripts (Albaugh et al., 2016; Lai et al., 2015; Shih et al., 2013) and the Analysis of Functional Neuroimages (AFNI) program. Region of interest (ROI) was placed on the dorsal hippocampus and primary somatosensory cortex to extract the time-course data. The ΔR_2_* values were calculated as follows: $\Delta {\text{R}}_{2}*=-\frac{1}{TE}$ ln [S(t)/mean(S_o_)], where S(t) represents the MR signal intensities over time and S_o_ represents the average signal intensity of baseline before CION injection. In order to accommodate for slight temporal delays due to manual CION injections, PK curves were aligned according to onset signal changes. To calculate various pharmacokinetic parameters, such as elimination constant (K_e_) and half-life (T_1/2_) of the contrast agent, we assumed that the maximum signal intensity change (ΔR_2_*_max_) caused by each CION formulation during the post-contrast injection period is directly proportional to the maximum (or peak) serum concentration that the contrast agent achieves in a specific compartment or test ROI. We assumed that the distribution and elimination of CION follows a one-compartmental model and accordingly, fit the decay of all contrast agents within a given ROI using a one-phase exponential decay model and ΔR_2_*_max_ as the initial value, *i.e. t* = 0. The pharmacokinetic profile of each CION was analyzed using the following equation:

$$Y=\left(Y_{0}-\;Plateau\right)*exp(-K\times t)+\;Plateau$$

where, Y_0_ is the Y value when t (time) is zero, Plateau is the Y value at infinite times, K is a rate constant expressed in reciprocal of the t axis time units. Except for CION_60000_, which decays to a non-zero, steady plateau within two hours of contrast-agent administration, the Plateau was constrained to 0 for CION_17143_, CION _12,000_, and Feraheme, each of which tends to reach *Y* = 0 at infinite time.

#### fMRI data.

All EPI data acquired for pharmacokinetic evaluation of CION were slice-time corrected, motion-corrected, skull-stripped, and registered to the template space using AFNI. For the hypercapnia experiment, we performed an independent component analysis (ICA) and compared ΔR_2_* derived between before and after the hypercapnic challenge. Signal before and after the initiation of the CO_2_ challenge was compared using a two-sample *t*-test based on the response pattern identified from ICA. For the visual and optogenetic stimulation studies, a general linear model (GLM) with third-order polynomial regression was used. Benjamini–Hochberg’s False discovery rate (FDR) correction procedure was applied for the multiple comparison (Benjamini and Hochberg, 1995). Finally, the imaging results have a threshold of *p* < 0.01 to demonstrate statistically significant clusters. To showcase functional relaxivity changes under CION, we did not normalize the signal to form percent CBV changes, but instead express all fMRI time-series data in ΔR_2_*.

#### μMRA data.

3D FLASH image volumes were spatially co-registered using AFNI to correct for any spatial drift between repetitions using AFNI and averaged to improve signal-to-noise-ratio (SNR). Vascular contrast was made by subtracting data before and after CION_17143_ administration. Brain were manually skull-stripped using ITK-SNAP and visualized using Amira (FE1, V5.3.3 or Avizo software, TGS, San Diego, CA, USA). Following masking only the blood vessels in the brain using Amira’s segmentation editor, a 3D-rendering of the cerebrovasculature was overlaid on anatomical T_2_-weighted images using Amira multi-plane viewer.

##### Toxicity, Biodistribution, and Histopathology.

To assess the biocompatibility of CION, toxicity studies were carried out in C57BL/6 J wild-type mice (*n* = 5). Mice were intravenously injected with CION at a higher 40 mg Fe/kg dose to examine biosafety in a wider dosing range. A separate group of mice (*n* = 5) was treated with Feraheme at the same 40 mg Fe/kg dose to serve as positive controls. General health and weight of animals were regularly monitored up to 7 days post-injection and humanely sacrificed with pentobarbital. All major organs such as liver, spleen, kidney, lungs and brain were immediately excised, submersion-fixed in 10% PFA for 72 h at 4 °C, processed on an ASP 6025 automated tissue processor (Leica Biosystems), and embedded in paraffin wax. Tissues were sectioned at 4 μm thick and mounted on glass slides. Tissue sections were deparaffinized prior to staining. Hematoxylin and eosin (H&E) staining was performed using pre-mixed hematoxylin, clarifier, bluing reagent, and eosin (Richard Allan Scientific). For Prussian blue staining, Gomori’s method for iron staining was used, employing a 1:1 hydrochloric acid-Potassium ferrocyanide solution made from 20% hydrochloric acid and 10% potassium ferrocyanide, and a nuclear fast red counter stain. Immunohistochemistry for CD45 was performed on the Discovery Ultra (Ventana Medical Systems) using manufacturer reagents. Antigen retrieval was performed using CC1 pH 8.5. CD45 antibody (BD Pharmigen, Cat # 550,539) was used at a final concentration of 1:2000 in Discovery PSS Diluent followed by anti-rat OmniMap HRP-conjugated antibody. Antigen detection was performed using Discovery Chromomap DAB, and nuclei were counterstained with Hematoxylin II and Bluing Reagent.

### Statistical analysis

All data unless otherwise specified are expressed as mean ± standard error of the mean. Statistical analysis was performed with Graph Pad Prism 8.30. The hydrodynamic sizes, PDI and ΔR_2_*_max_ of various CION formulations were compared using a one-way analysis of variance (ANOVA). Multiple comparison tests were performed using Tukey-Kramer HSD post-hoc analyses. To compare the contrast properties between a pair of CION formulations at different time points, the Δ*R*_2_* values of a given formulation corresponding to baseline, as well as multiple post-injection periods were averaged. We performed two different types of comparison: (1) within group comparison to evaluate the averaged Δ*R*_2_* across different time points of a given formulation; and (2) inter-group comparison to compare the Δ*R*_2_* of different formulations at a particular time interval. Statistical analysis was performed using a two-way ANOVA, followed by Tukey’s multiple comparison tests. *p* < 0.05 was used to denote statistical significance.

## Results

### CION synthesis and characterization.

**Fig. 2.**
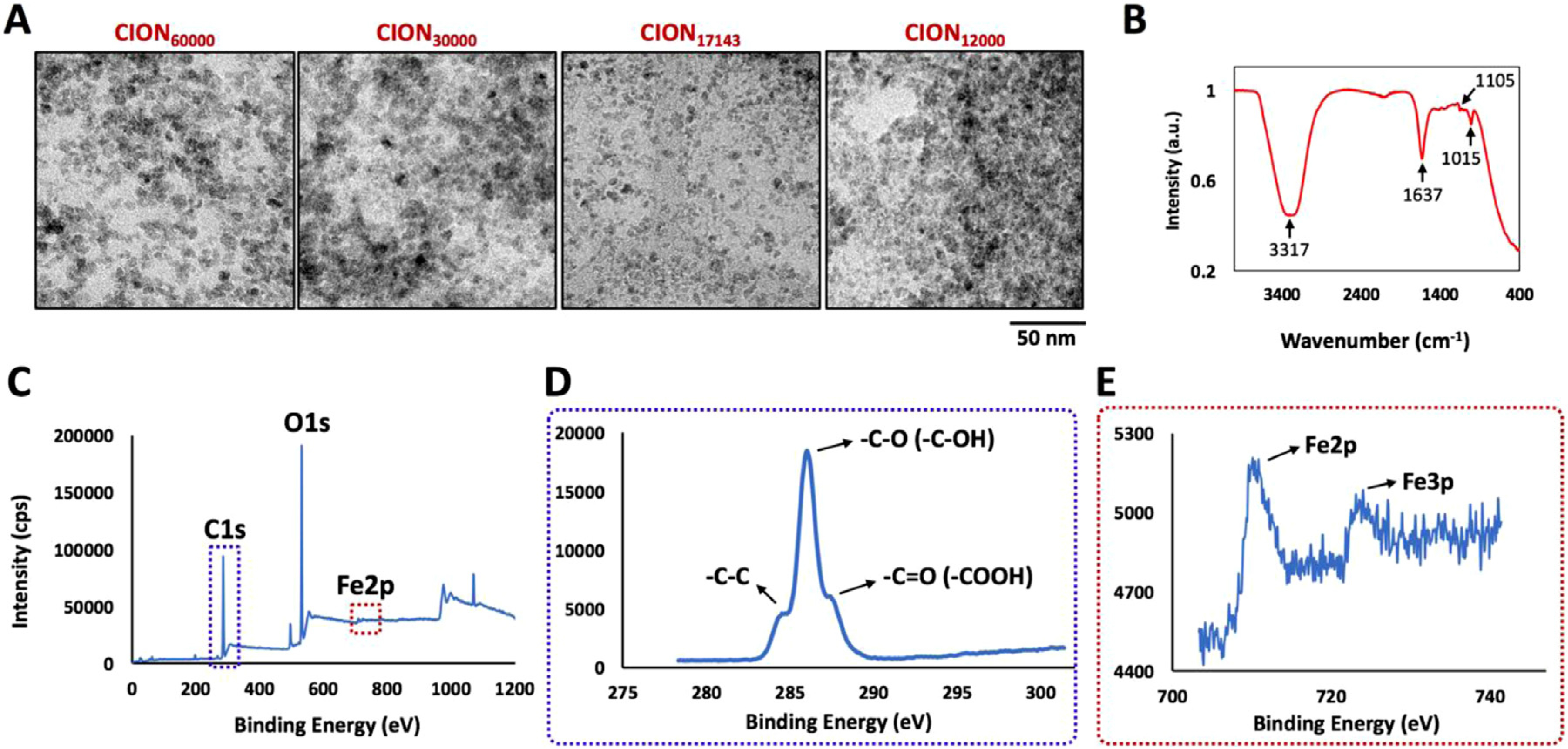
Physicochemical Characterization of CION. **(A)** Representative high-resolution TEM images of CION_60000_, CION_30000_, CION_17143_ and CION_12000_. **(B)** ATR-FTIR spectrum of CION_17143_. **(C)** Full XPS spectrum for CION_17143_ and isolated high-resolution XPS scans of **(D)** C1s and **(E)** Fe2p peaks.

The synthesis of CION has been schematically illustrated in Fig. 1 and further detailed in Video S1. We observed that CION synthesized with the molar ratio of Fe:CMD above 60,000:1 (assuming molecular weight of CMD = 10 kDa) were prone to agglomeration and therefore did not consider evaluating any higher ratios. In contrast, all nanoformulations synthesized with Fe:CMD ratio equal to or lower than 60,000:1 were stable in PBS with no visual aggregation for at least one month. The hydrodynamic diameter and PDI of CION synthesized with different Fe:CMD molar ratio are presented in Table S2. As the molar stoichiometric ratio of Fe:CMD decreased from 60,000:1 to 17,143:1, a statistically significant (*p* < 0.0001) decrease in the hydrodynamic size from 365.68 ± 5.04 nm to 50.65 ± 2.49 nm was observed. Concomitantly, a significant (*p* < 0.01) decrease in PDI from 0.423±0.021 to 0.240±0.018 was noted. Further reduction in Fe:CMD ratio (*e.g.* 12,000:1) had no significant effect on the PDI of nanoparticles though a notable (*p* < 0.0001) increase in hydrodynamic size occurred (96.30 ± 2.67 nm). Among all nanoformulations, CION_17143_ presented the smallest hydrodynamic size ranging between 45 and 60 nm. We tested 4 different batches for reproducibility assessment and found the mean hydrodynamic diameter of the nanoparticles was 50.65 ± 2.49 nm and mean PDI was 0.2406±0.037 (Fig. S1). This nanoformulation did not show any visual aggregation in PBS for at least 6 months within a concentration of 10–30 mg Fe/ml. Fig. 2A presents the high resolution TEM images of CION_60000_, CION_30000_, CION_17143_ and CION_12000_. In all cases, nanoparticles were spherical or quasi-spherical in shape with particle size ranging between 5 and 10 nm. The electron dispersive X-ray spectrum of CION_17143_ (Fig. S2) confirmed the presence of Fe, C and O in the sample. The ATR-FTIR spectrum of CION_17143_ (Fig. 2B) displayed characteristic peaks at around 1105 cm^−1^ and 1015 cm^−1^, ascribed to the presence of C–O bonds, and *α*-glucopyranose ring associated with the surface-dextran coat. The presence of prominent stretching vibration band at 1637 cm^−1^ was indicative of the presence of carboxylic acid groups (−COOH) on the NP surface. Additionally, a broad band was observed in the frequency range of 3000–3500 cm^−1^, ascribed to the −OH stretching vibration of −COOH and −OH functional moieties associated with the CMD surface-coat on iron-oxide. A typical survey XPS spectrum of CION_17143_ is presented in Fig. 2C. The survey scan revealed the presence of C1s, O1s, Fe2p and Fe3p peaks at binding energy values of 285, 531, 710 and 725 eV. The high resolution C1s spectrum of CION_17143_ (Fig. 2D) showed three distinct peaks at 285, 287 and 288 eV corresponding to the C–C, C–O and *C* = *O* bonds inherent to chemical backbone of CMD present on the surface of CION. The Fe2p doublets with binding energy values of 710 and 725 eV (Fig. 2E) were representative of Fe-O bonds, typical of magnetite. We also used XPS to quantify the atomic concentrations of Fe, O and C on the surface of CION. Our results indicated that increasing the amount of polymer during co-precipitation reaction led to an increase in surface C:Fe atomic ratio (Fig. S3, Table S3).

### *In vitro* relaxometry.

**Fig. 3.**
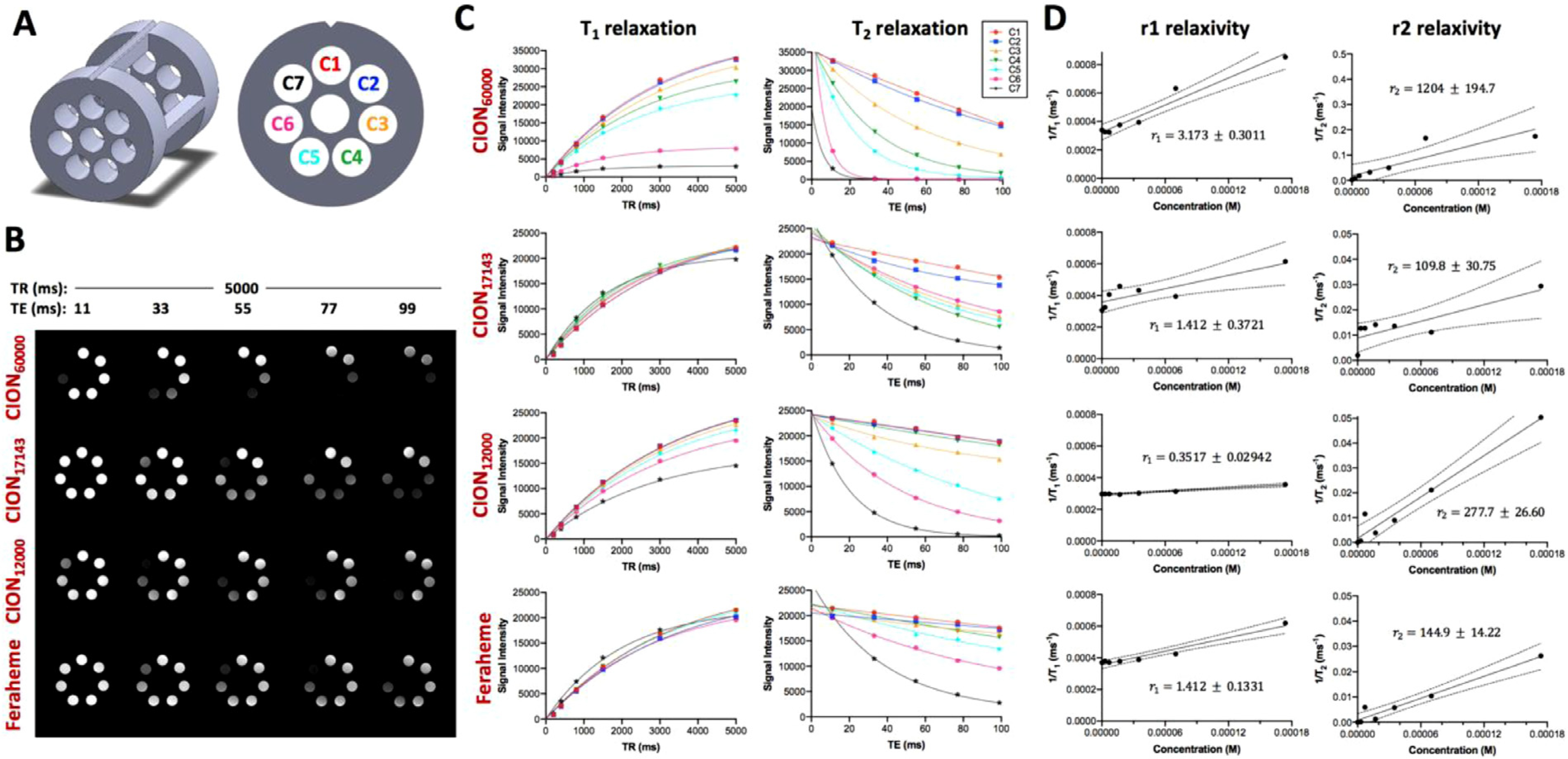
*In vitro* relaxometric analysis of CION. **(A)** Custom-design holder for relaxometric analysis. **(B)** Representative T_1_- and T_2_-weighted MR phantom images of various CION compositions and Feraheme acquired at different TR and TE. Note that this experiment collected a range of TR (5000, 3000, 1500, 800, 400 and 200 ms) and TE (11, 33, 55, 77 and 99 ms), but the figure only displays varying TE images with the longest TR due to space constraint. **(C)** T_1_ and T_2_ relaxation plots. **(D)** Quantification of r_1_ and r_2_.

RepresentativeMR phantom images of CION_60000_, CION_17143_, CION_12000_ and Feraheme acquired at different TRs and TEs using a custom-built holder (Fig. 3A) showed a concentration-dependent negative contrast enhancement when compared to saline control (Fig. 3B). The T_1_ and T_2_ relaxations of various CION compositions are presented in Fig. 3C. Among all the formulations tested, CION_60000_ exhibited the highest r_2_/r_1_ ratio (r_1_ = 3.173 mM^−1^s^−1^; r_2_ = 1204 mM^−1^s^−1^) at 9.4T, followed by CION_12000_ and CION_17143_. The relaxivity of CION_17143_ (r_1_ = 1.412 mM^−1^s^−1^; r_2_ = 109.8 mM^−1^s^−1^) was comparable to that of Feraheme (r_1_ =1.412 mM^−1^s^−1^; r_2_ = 144.9 mM^−1^s^−1^) measured in this study as well as dextran-coated monocrystalline iron oxide nanoparticles (MION) reported by Zhao et al. at 9.4T (r_1_ = 1–1.7 mM^−1^s^−1^; r_2_ = 70–110 mM^−1^s^−1^) (Zhao et al., 2003).

### In vivo Pharmacokinetics and Clearance.

**Fig. 4.**
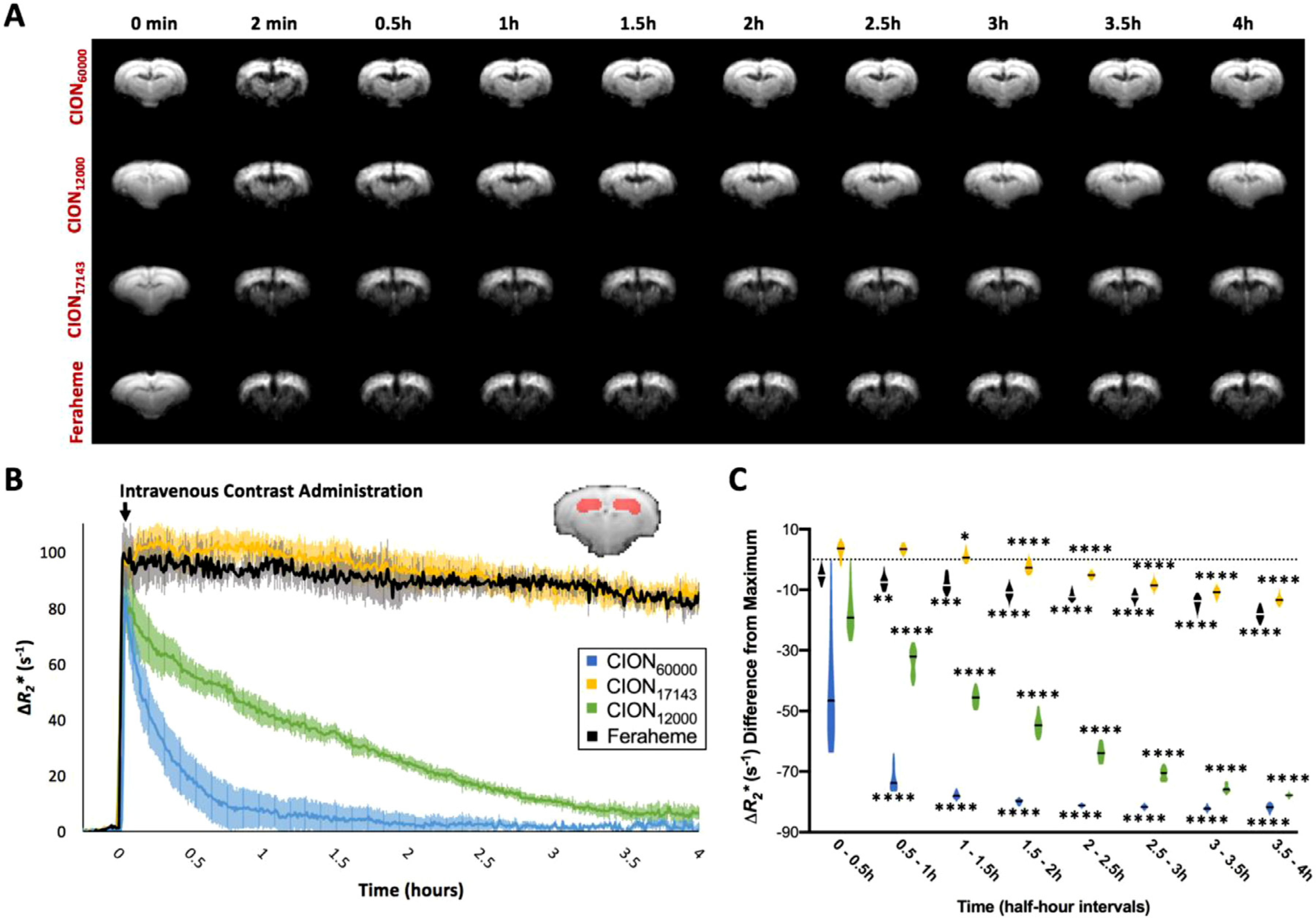
*In vivo* pharmacokinetic evaluation of CION conjugates and Feraheme in the rat brains. **(A)** Group-averaged EPI images of rat brains at various time points after CION_60000_ (*n* = 3), CION_17143_ (*n* = 6), CION_12000_ (*n* = 4) and Feraheme (*n* = 3) administration. **(B)** Hippocampal Δ*R*_2_* signal changes were evaluated up to 4 h following administration of each CION conjugate and Feraheme. **(C)** Δ*R*_2_* signal decay from maximum contrast were calculated for every half-hour period and statistically compared to the first half-hour period for each contrast agent. *denotes *p*<0.05, **denotes *p*<0.01, ***denotes *p*<0.001, ***denotes *p*<0.0001.

Fig. 4A presents the *R*_2_* signal change profile of CION_60000_, CION_17143,_ CION_12000_ and Feraheme on EPI scans of the rat brain. A two-way ANOVA examining the effect of all contrast agents on Δ*R*_2_* signal change in the hippocampus in 30 min intervals compared to baseline found a statistically significant interaction between the effects all contrast agents and the enhancement of Δ*R*_2_* signal compared to pre-contrast baseline (F_(3, 324)_ = 216.6, *p* < 0.0001) in the 30 min following tail vein administration (Fig. 4B and C). CION_12000_ and CION_60000_ displayed rapid intravascular clearances with respective circulating half-lifes of 60.5 min and 14 min. CION_60000_ had a rapid, one-phase exponential decay with Δ*R*^2^* reaching near baseline levels within 170 min of administration. In contrast, CION_17143_ displayed an ideal pharmacokinetic profile for steady-state applications. A bolus, intravenous injection of CION_17143_ increased the maximum Δ*R*_2_* to 102.342 ± 5.937 *s*^−1^ following administration, which was comparable to the maximum Δ*R*_2_* of Feraheme (*p* = 0.0636). Tukey-Kramer HSD *post-hoc* analysis indicated that Δ*R*_2_* change following CION_17143_ administration remained statistically unaltered for the first hour (*p* = 0.0556). Using a one-phase, exponential decay model, the half-lives of CION _17,143_ and Feraheme were calculated to be 472.3 min (7.87 h) and 468.4 min (7.81 h), respectively (Fig. S4). Because the CION_17143_ and Feraheme exhibited linear-like pattern of clearance, we also fit the clearance of both formulations using a linear regression and their slopes were found to statistically comparable (*p* = 0.2186; Fig. S5). Due to its long intravascular half-life, CION_17143_ was selected for subsequent *in vivo* fMRI evaluations. The derivation of various pharmacokinetic parameters such as elimination constant (K_e_) and half-life (T_1/2_) from are shown in Fig. S4. Additionally, we also measured the Δ*R*_2_* signal change profile for CION_60000_, CION_17143,_ CION_12000_ and Feraheme in the rat S1 cortex (Fig. S6).

### *In vivo* fMRI applications.

**Fig. 5.**
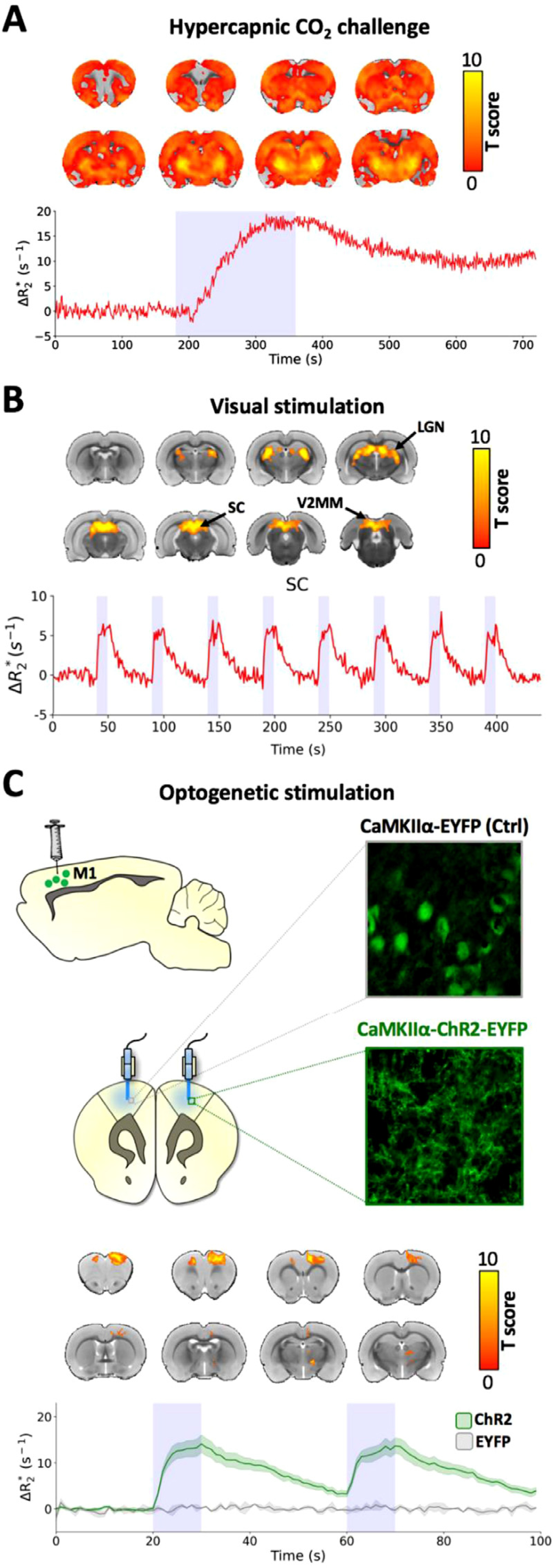
*In vivo* fMRI applications of CION_17143_ in rats. **(A)** CBV activation map and global signal time-course Δ*R*_2_* changes in response to hypercapnia. **(B)** Visual stimulation induces CBV activation along brain regions of the visual network and time-locked Δ*R*_2_* changes in the SC in response to visual stimulation epochs (responses from additional ROIs shown in Supplemental Fig. S7). **(C)** Viral transfection of the control EYFP construct (left M1) produced a relatively uniform EYFP expression pattern consistent with the cytoplasmic cell-filling properties of the protein, while transfection of ChR2-EYFP (right M1) resulted in a more diffuse EYFP expression pattern consistent with the trafficking and transmembrane characteristics of ChR2. CBV activation map and M1 time-course Δ*R*_2_* changes in response to optogenetic stimulation. Threshold applied at *p* < 0.05 with FDR correction.

We selected well-established fMRI procedures (Chan et al., 2017; 2012; Keilholz et al., 2006; Leong et al., 2016; Liang et al., 2015; Lin et al., 2008, 2009; Schmid et al., 2017, 2016; Wu et al., 2003; Yu et al., 2016; Zhao et al., 2005, 2006) to demonstrate the feasibility of using CION_17143_ for fMRI with no intention to power the experiment to address any novel biological effects. Fig. 5 shows the use of CION_17143_ in several common stimulus evoked-fMRI paradigms, including hypercapnic CO_2_ challenge, visual stimulation, and targeted optogenetic stimulation. With the steady-state created by 20 mg/kg of CION_17143_, 5% CO_2_ challenge induced Δ*R*_2_* increases throughout the brain, with the changes up to 15 s^−1^ (Fig. 5A). It should be noted that the CO_2_-induced CBV-fMRI signal changes exhibited delayed onset and offset, likely attributed to the dead volume in the ventilator module or CO_2_ delivery tubing as well as the time needed for the cerebral blood gas to respond to the different inhaled gases (*i.e.*, CO_2_ or medical air). Visual stimulation at 10 Hz induced time-locked Δ*R*_2_* increases throughout the rat visual network with robust activity in the superior colliculus (SC; Fig. 5B) and lateral geniculate nucleus (LGN; Fig. S7) with minimal activation in the mediomedial area of the secondary visual cortex (V2MM; Fig. S7). Optogenetic stimulation at 40 Hz induced Δ*R*_2_* increases by 10 *s*^−1^ in the targeted primary motor cortex (M1) where Channelrhodopsin-2 (ChR2) was virally expressed (Fig. 5C).

### High resolution μMRA.

**Fig. 6.**
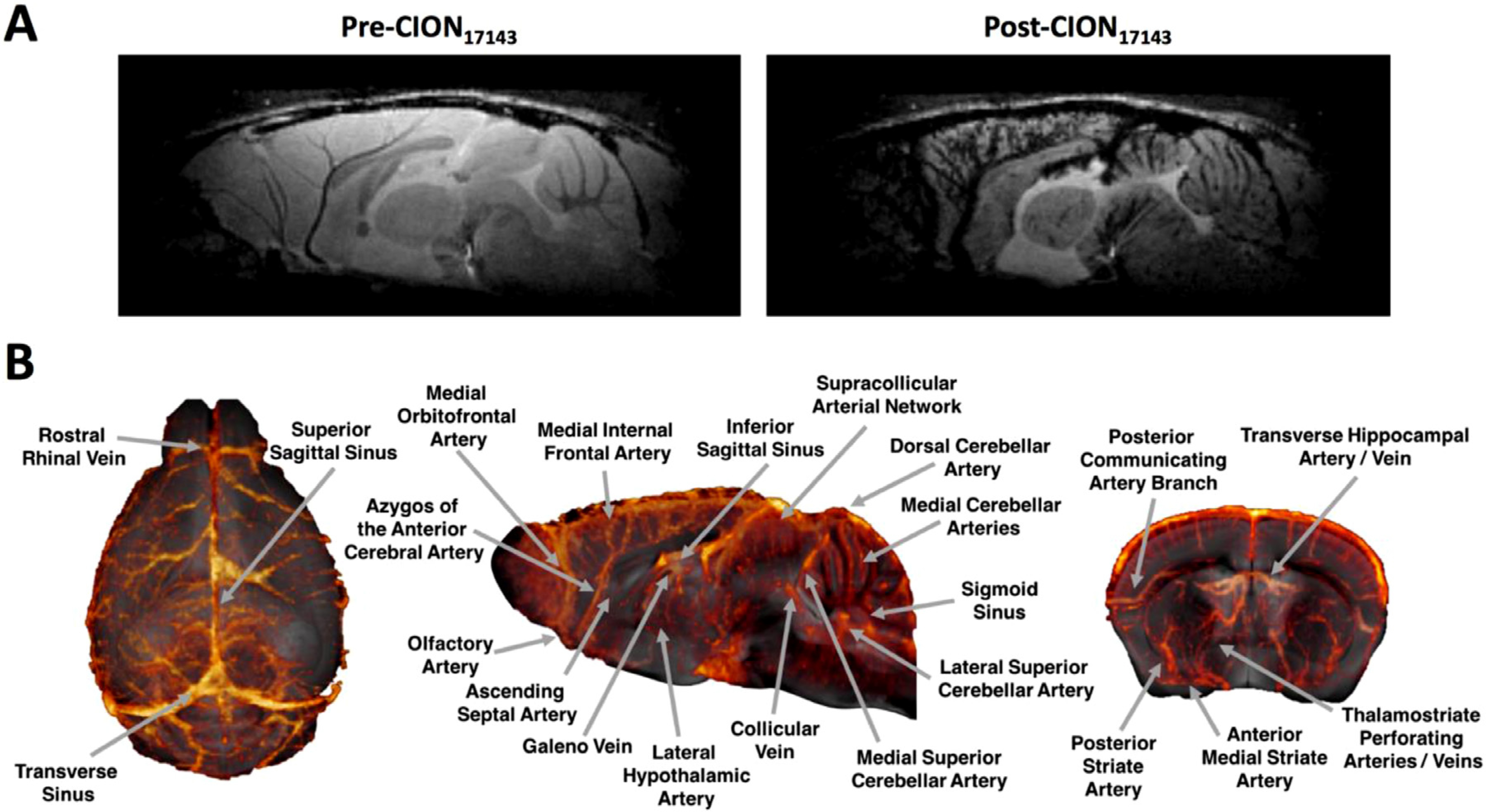
MR angiography using CION. **(A)** FLASH-3D images of mouse brain captured at 100 μm^3^ spatial resolution before and after injection of CION_17143_. **(B)** Representative 3D cerebrovasculature of the mouse brain generated from a post- and pre-contrast of FLASH 3D images acquired at 50 μm^3^ spatial resolution.

To determine the utility of CION_17143_ as a μMRA contrast agent, we performed a proof-of-concept experiment in a mouse. Fig. 6A presents the pre-and post-contrast FLASH-3D images of a mouse brain imaged at 100μm^3^ spatial resolution. The post-contrast image revealed the presence of several cerebral vessels in the cortical and subcortical regions not clearly visible prior to CION_17143_ injection. Our findings identified similar vasculature observed in other μMRA studies using a similar USPIO-enhanced methodology (Fouquet et al., 2020; Klohs et al., 2012; Lin et al., 2013). The long intravascular half-life of CION_17143_ enabled the acquisition of images at higher spatial resolution (50 μm^3^ isotropic for the provided example). Fig. 6B shows the representative 3D cerebrovasculature of the mouse brain. We were able to identify several key arteries and veins from this dataset including rostral rhinal vein, galeno vein, collicular vein, transverse hippocampal arteries/veins, thalamostriate perforating arteries/veins, medial internal frontal artery, supracollicular arterial network, dorsal and medial cerebral arteries, lateral superior cerebella arteries and anterior medial striate artery.

### Fluorescent-tagged CION for multimodal application.

**Fig. 7.**
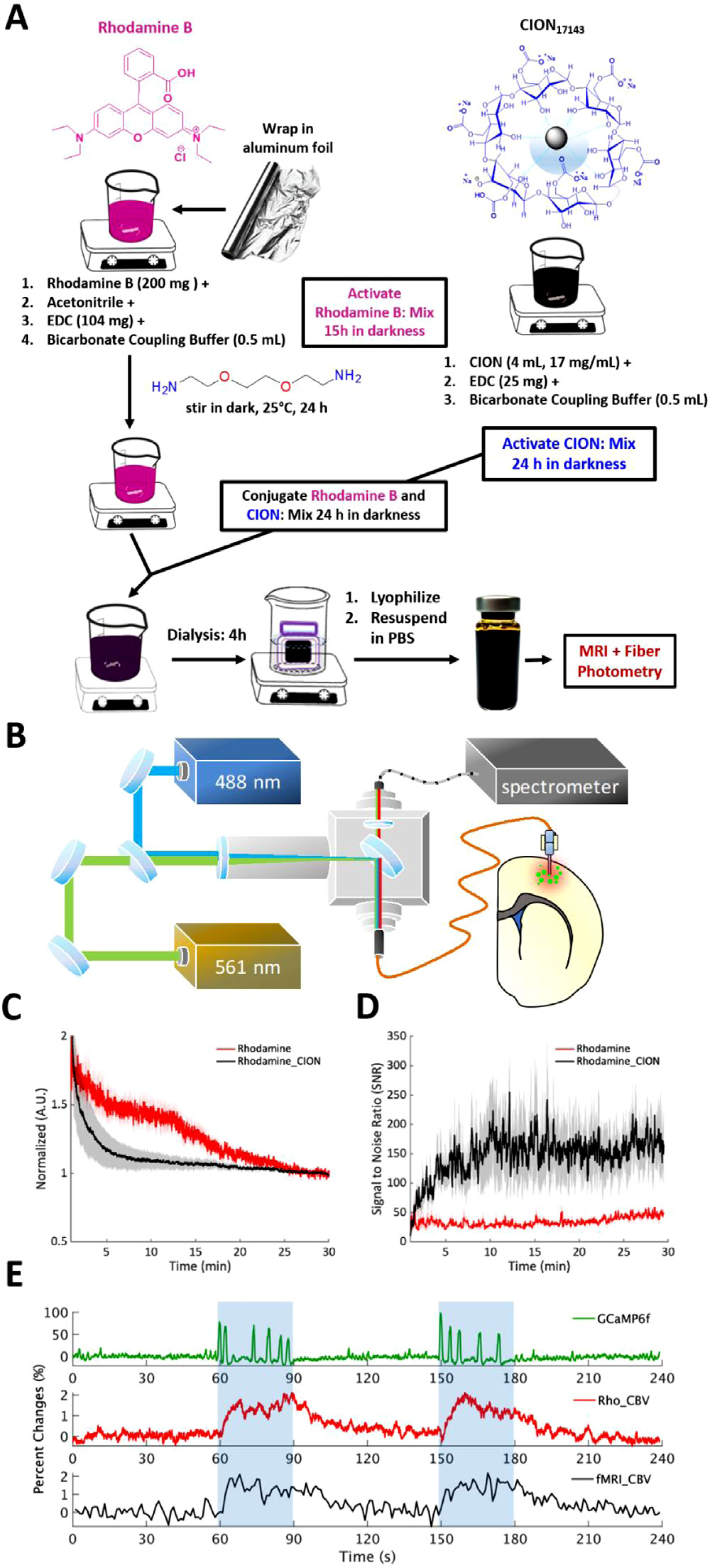
Concurrent fMRI-photometry application using fluorescent-labeled CION. **(A)** A schematic methodology depicting how CION_17143_ is conjugated with the fluorescent Rhodamine B. **(B)** Fiber-photometry system uses two lasers to excite GCaMP (*λ*=488 nm) and Rhodamine (*λ*=561 nm), respectively, and a spectrometer to detect emitted photons changes over time. **(C)** Intravascular clearance measured by fiber-photometry (*n* = 4/contrast). Rhodamine-CION conjugate (black) shows faster stabilization to a steady-state compared to Rhodamine B (red). Note that the Y-axis represents a normalized intensity, thus a higher values of Rhodamine B does not represent a stronger signal. **(D)** Signal-to-noise-ratio (SNR) of Rhodamine-CION conjugate is higher compared to Rhodamine B (*n* = 4/contrast). Error bars are standard error of the mean. Note that the Rhodamine-CION group shows a larger standard error, likely because it represents inter-subject variability. Indeed, the ratio of SNR and standard error was more comparable between Rhodamine and Rhodamine-CION groups. It should be noted that spontaneous activity fluctuation could also be amplified in Rhodamine-CION measurement, causing a larger SNR changes. **(E)** Concurrent fMRI and fiber-photometry recording (10 Hz sampling rate) shows increases in neuronal activity reflected by intercellular calcium release as well as CBV changes by both Rhodamine-CION conjugate fluorescence and fMRI-CBV measurement during forepaw electric stimulation (blue shading, 3 mA, 9 Hz, and 0.5 ms pulse width). (For interpretation of the references to color in this figure, the reader is referred to the web version of this article.).

To demonstrate the utility of CION platform for cross-modality application, we conjugated CION with Rhodamine B, a red-fluorescent dye, using the protocol shown in Fig. 7A and Supplementary Materials, and administered this conjugate intravenously via tail vein in rats. Fig. 7B shows the photometry experimental setup and Fig. 7C shows photometry-measured normalized pharmacokinetic time-courses of Rhodamine-CION conjugate within 30 min after the injection and compares with injection of Rhodamine B alone (red) in the same animal with a few days apart. Notably, Rhodamine-CION appeared reaching a steady state faster than Rhodamine B alone. Rhodamine-CION also exhibited higher signal to noise ratio (SNR) than Rhodamine B (Fig. 7D). These data indicated that the conjugate was feasible, and Rhodamine-CION helped stabilize the pharmacokinetics, which should allow better CBV measurement using optical methods. To demonstrate a possible cross-modality application, we conducted simultaneous fMRI and fiber-photometry recording using the conjugated Rhodamine-CION with conventional forepaw electrical stimulation. Similar to prior *in vivo* application studies, we chose a well-established protocol to showcase the feasibility with no intention to address any novel biological effects thus did not fully power the experiment. Fig. 7E showed that GCaMP6f (neuronal calcium activity) and Rhodamine-CION (CBV) both increased during the stimulation period, and the CBV-fMRI time-course concurrently measured by Rhodamine-CION showed highly consistent pattern with the photometry CBV signal.

### *In vivo* biocompatibility.

To study the biocompatibility of CION_17143_, we examined toxicity using histology. CION_17143_ was administered intravenously in mice at a higher dose of 40 mg Fe/kg and the results were compared with that from a separate group of mice receiving Feraheme injection at the same dose. As a preliminary step towards toxicity evaluation, we monitored the general health conditions of mice up to 7 days post-injection. We found no significant changes in body weight during the post-injection period (Fig. S8). While we did not systematically measure behavioral metrics in this study, our facility veterinary technicians on duty reported no pain or sickness behavior following CION_17143_ treatment. All animals were sacrificed for histological assessment on day 7 after CION_17143_ injection. H&E and Prussian blue staining, as well as CD45 labeling was performed on subsequent tissue sections to assess representative organ histopathology. The data were reviewed by a board-certified veterinary pathologist (S.A.M.) blinded to the treatment groups at time of analysis. As evident from both H&E as well as CD45 staining (Fig. 7), livers from both groups displayed mild lymphocytic inflammation, with the CION_17143_ treated group displaying comparable, if not less inflammation than the Feraheme-treated group. Livers from both groups displayed similar amounts of extramedullary hematopoiesis, a common, nonspecific finding in mice. H&E findings of the kidney, spleen, brain, and lung in both groups exhibit normal feature. Prussian blue staining of the kidney revealed occasional iron deposition within the nephron, suggestive of renal filtration of the compounds. Prussian blue staining of spleens in both groups revealed moderate-numbers of iron-laden macrophages, which is expected in the spleen (a site of erythrocyte turnover) and is not ascribed to the treatment regimes.

## Discussion

Over the past several years, many studies have proposed long-circulating, blood pool MR contrast agents (Zhou et al., 2017) synthesized by coating SPIO/USPIO with dextran or carboxymethyl dextran derivatives (Ayala et al., 2013; Creixell et al., 2010; Li et al., 2011; Liu et al., 2011; Sharma et al., 2018; Tassa et al., 2011; Wunderbaldinger et al., 2002; Groman et al., 2003), PEG-(Khandhar et al., 2017), PEG-derivatized phosphine oxide (Kim et al., 2011), PEGylated-silica (Wu et al., 2011) and bisphosphonate-anchored PEG (Sandiford et al., 2012). Many of these formulations such as crosslinked iron-oxides (CLIO) developed by Weissleder (Wunderbaldinger et al., 2002) or PEGylated-silica coated iron oxide nanoparticles developed by Wu et al. (2011) have shown promise for preclinical MRI application including cerebrovascular and cardiovascular imaging. However, these syntheses are based on a rather complex workflow that involves multi-step reactions with tedious purification steps and/or costly equipment and reagents, posing reproducibility and scaling-up challenges for laboratories lacking high-end chemistry resources. This study provides a detailed step-by-step guideline on how to synthesize an iron-oxide based MRI contrast agent from scratch using a streamlined and affordable protocol (Table S1). We also provide steps to tune the physicochemical properties of CION so that it can be made suitable for different types of *in vivo* applications. The simplicity of our one-pot method allows researchers to reproduce this method in their laboratories easily and tailor their nanoparticles according to their imaging needs by controlling reaction variables. For instance, CION_17143_ is an ideal choice for steady-state CBV assessment due to its long intravascular half-life. However, the short-circulating CION_60000_ may be best suited for applications such as imaging of liver, spleen, tumor. It might also find useful when selective targeting is needed for the contrast agent to flush out rapidly from the vascular compartments, leaving only targeted agent bound to disease-specific biomarkers. It is our hope that making our recipe publicly available will benefit chemists and MRI researchers to further advance the field.

To identify the optimal reaction conditions that can lead to maximum CMD grafting on nanoparticles while still having a decent iron payload per particle, a series of nanoformulations were formulated with varying stoichiometry of Fe to CMD. As observed by DLS, a decrease in Fe:CMD molar ratio from 60,000:1 to 17,143:1 led to a diminution in hydrodynamic size from 365.36 ± 5.04 nm to 50.65 ± 1.00 nm due to the increased polymer coating associated with nanoparticle surface, enhancing steric and electrostatic stabilization. But after a certain concentration of CMD in solution, the trend was reversed. Subsequently, a decrease in Fe: CMD molar ratio from 17,143:1 to 12,000:1 increased the nanoparticle size to 96.30 ± 2.67 nm. This observation is not surprising and may be attributed to depletion flocculation which occurs when the amount of polymer in the solution exceeds the threshold required to completely cover the surface of nanoparticles. When the excess polymer exists in the reaction mixture and the fully coated nanoparticles approach closer (*i.e.* less than the radius of gyration), the non-adsorbed free polymers between them are subject to exclusion due to entropic effects. Subsequently, the local concentration becomes lower than the surrounding level. The difference in concentration leads to an osmotic pressure difference, attracting two particles and eventually increasing the aggregate size. The TEM image of all nanoformulations prepared with Fe:CMD ratio between 60,000:1 to 12,000:1 showed hundreds of ultrafine, spherical to quasi-spherical particles with diameter ranging between 5 and 10 nm. Unlike DLS, an increase in the amount of coating agent during synthesis had nominal impact on the core size of nanoparticles. For all nanoformulations, particle sizes measured in TEM were at least 10–15 times smaller than their corresponding hydrodynamic sizes. This difference can be attributed to the differences in underlying principles involved in size measurement using these techniques. While TEM is used to image dry nanoparticles, DLS measures the size of hydrated nanoparticle, which include the contribution of the surface coating associated with the nanoparticle core and their solvation in the dispersion medium. The surface chemistry of CION was studied using ATR-FTIR spectroscopy. All tested nanoformulations (*i.e.* CION_60000_, CION_17143_ and CION_12000_) presented identical spectral pattern except for increasing C–O stretching vibration intensity at around 1105, and 1015 cm^−1^. The intensity of these bands can be qualitatively related to CMD functionalization density associated with the iron oxide core as we observed a smaller signal intensity for CION_60000_ and a stronger intensity band for CION_12000_ possessing a higher polymer content. We also observed a strong stretching vibration at 1637 cm^−1^ band, which was ascribed to the presence of carboxyl functionality associated with the CMD coat. This band, however, undergoes a hypsochromic (blue) shift when compared to the expected stretching region of carboxyl group, which is between 1700 and 1725 cm^−1^. This blue shift, in part, could be contributed by the complexation of carboxyl groups on metal surface and/or presence of extensive hydrogen bonding between surface carboxyl groups. The presence of CMD coating on the surface of CION was further confirmed using XPS. As evident from the high resolution C1s XPS spectrum of CION_17143,_ these NPs contained reactive carboxyl groups on their surface to allow further modification with functional biomolecules including proteins, antibodies, aptamers and therapeutic drugs. A thorough analysis of surface functionality and elemental composition using XPS conferred that the C:Fe atomic ratio on nanoparticles critically depended on the stoichiometric ratio of metal precursors to polymer used during nanoparticle synthesis.

To examine how variation of Fe:CMD molar ratio affects the relaxivity of nanoparticles, we measured the r_1_ and r_2_ for various CION compositions, namely, CION_60000_, CION_17143_ and CION_12000_ using a conventional spin-echo based T_1_ and T_2_ mapping protocol. Among all the formulations, CION_60000_ exhibited the highest r_2_/r_1_ ratio, followed by CION_12000_ and CION_17143._ Two factors that determine the contrast enhancement of CIONs might help explain our results: (1) iron-oxide core (size and material); (2) thickness (coating and chemical composition). SPIO and USPIO are known to shorten T_2_, which is governed by the translational diffusion of H_2_O molecules in the inhomogeneous magnetic field surrounding the nanoparticles (Gillis and Koenig, 1987). According to quantum-mechanical outer sphere theory, the T_2_ relaxivity of CION in solution can be expressed by the following equation:

$$1/T_{2}=\left(256\pi^{2}\gamma^{2}/405\right)V*M_{s}^{2}a^{2}/D(1+L/a)$$

where *γ* denotes proton gyromagnetic ratio; V*, M_s_ and a represented the volume fraction, saturation magnetization and the radius of iron oxide core, respectively; D is the diffusivity of water molecules, and L is the thickness of an impermeable surface coating (Brooks et al., 2001; Gillis et al., 2002; Tong et al., 2010). Of note, the coating molecules associated with iron-oxide nanocore can result in exclusion of water from its surface, prevent water diffusion, or immobilize surrounding water molecules through formation of hydrogen bonds (Tong et al., 2010); all these factors can affect the nuclear relaxation of water protons. In this case, as the Fe:CMD ratio was lowered from 60,000:1 to 17,143:1, the hydrodynamic size of CION decreased with a concomitant increase in CMD coating thickness on the nanoparticle surface, resulting in a lower r_2_/r_1_ ratio, which was slightly less than Feraheme but greater than MION. However, a further increase in the amount of polymer during CION synthesis, increases the r_2_/r_1_ of CION. This observation, albeit off-the-trend, can be explained by an increased polymer coating thickness, which is supposed to have a deleterious effect on the saturation magnetization of the iron-core.

We evaluated the pharmacokinetic properties of three representative CION compositions – CION_60000_, CION_17143_ and CION_12000_. Although intravenous injection of CION_60000_ in rats led to reasonable contrast enhancement *in vivo*, our preliminary data indicated the half-life of this agent (11.2 min) was too short to meet prolonged imaging requirements, critical for several functional neuroimaging applications. This fast-clearing CION, however, can be well-suited for studies that have a shorter acquisition window such as liver and spleen imaging, cell-labeling and tumor targeting where fast clearance is essential to have unbound contrast agent removed from the vessels eliminating unnecessary background signal. Among all three formulations, CION_17143_ presented the best results with regards to circulation stability (T_1/2_ ~ 10 h), while maintaining reasonable T_2_* contrast *in vivo*. Our results indicated that the r_2_ and intravascular half-life of CION_17143_ is comparable to Feraheme, which makes it an ideal contrast agent for steady-state MRI applications. These results suggest that the molar ratio should not be too big or small to achieve long half-life, and an intermediate ratio (such as 17,143:1) could yield smaller hydrodynamic size and hence longer intravascular circulation.

We presented a few proof-of-concept fMRI studies. These studies were not intended to test the efficacy of our contrast agent in a large cohort and address specific neuroscience questions. Among these studies, hypercapnia or an elevated pCO_2_ acts as a potent stimulator for vasodilation throughout the brain (Donahue et al., 2009; Faraci et al., 1994; Lu et al., 2009; Madden, 1993; Pollock et al., 2009; Yezhuvath et al., 2009), while visual stimulation in rodents increased activity in LGN and SC with variable responses ranging from increased to suppressed activation in visual cortices depending on the study design (Dinh et al., 2021; Jin and Kim, 2008a; Kim and Kim, 2011; Niranjan et al., 2016; Zhao et al., 2006). Using CION_17143_, we were able to detect robust, site-specific Δ*R*_2_* changes in rats exposed to visual stimulation and hypercapnic challenge under lightly anesthetized conditions similar to those reported by others (Brevard et al., 2003; Dinh et al., 2021; Jin and Kim, 2008a; Lu et al., 2009; Niranjan et al., 2016). CION_17143_ also demonstrated success in detecting robust optogenetic fMRI response in the cortical areas expressing ChR2 as expected (Lee et al., 2010; Ryali et al., 2016). Together, these results confirmed the efficacy of CION_17143_ for fMRI applications. Due to its long plasma half-life and robust T_2_ effects, this nanoformulation also showed excellent performance as a μMRA contrast agent, allowing improved visualization and delineation of cerebrovasculature *in vivo*. To validate the efficacy of CION for simultaneous fMRI and optical fiber photometry, we conjugated CION with a red fluorescent dye, Rhodamine B. The presence of reactive carboxyl groups enabled facile immobilization of Rhodamine B on the surface of CION through a short, PEG-type spacer – 2,2′-(ethylenedioxy)-bis-(ethylamine) (EDEA) using well-established carbodiimide chemistry (Li et al., 2018). Although further characterizations are required to confirm the chemical structure of CION-rhodamine conjugate, our preliminary cross-modality experiments suggested that Rhodamine-labeled CION could serve as a platform for bridging tools across different spatiotemporal scales.

To determine the biocompatibility of CION_17143_, we performed toxicity study in mice and compared the results with Feraheme. For toxicity analysis, we used a dose (40 mg/kg) that was higher than the highest dose (30 mg/kg) typically used for CBV-fMRI studies (Albaugh et al., 2016; Decot et al., 2017; Lai et al., 2015; Shih et al., 2013). We did not observe any abrupt changes in body weight up to 7 days post-injection; animals from both treatment group were apparently healthy and resumed their normal behavior and activities. Although H&E staining revealed the presence of mild lymphocytic inflammation in the livers from both treatment groups, the CION-treated group displayed slightly lower inflammation than Feraheme. For both treatment groups, no abnormalities were observed in the histopathological sections of kidney, spleen, brain and lung. To examine the biological fate of CION after 7 days post-administration, histopathological sections of liver, spleen, kidney, lungs and brain were also stained with Prussian blue, a common stain to detect the presence of iron in tissue specimens. Although we did not observe any iron accumulation in brain or lungs, iron deposits were visible in liver and spleen. This distribution profile is consistent with the proposed mechanism of iron metabolism for USPIO (Daldrup-Link, 2017). These results further indicate that both CION and Feraheme are eventually cleared from the intravascular space and enters the liver and spleen where they are most likely cleared by macrophages of the reticuloendothelial system (RES). As previously reported, it is possible that the nanoparticle coating is cleaved by lysosomal enzymes and the iron core is incorporated into the body’s iron stores, where it is metabolized over 6–12 weeks. (AMAG Pharmaceuticals, 2017) Prussian blue staining of kidney also revealed minimal iron deposition within the nephron, indicating renal excretion of the nanoformulations. In summary, our results suggest that CION_17143_ is a robust, reproducible, and safe steady-state contrast agent for preclinical MRI applications.

## Conclusion

We developed a simple, inexpensive method to synthesize high-performance intravascular CION for MRI applications. By tuning the material and pharmacokinetic properties of CION through careful surface modulation, we formulated a blood pool agent that offers robust and stable contrast for high resolution CBV anatomical and functional imaging. Additionally, we developed and characterized other CION variants with shorter circulation half-life and high r_2_/r_1_ ratio; although these compositions cannot be used for steady-state imaging, they hold significant promise for liver or spleen imaging, cell labeling and/or tumor targeting *in vivo.* Finally, CION possesses a reactive carboxyl handle that enables conjugation with a variety of functional biomolecules and imaging entities. By labeling CION with a red fluorescent dye, Rhodamine B, we demonstrated the effectiveness of CION for simultaneous fMRI and optical fiber-photometry. Our future studies will focus on further optimization of CION conjugate for various multimodal studies and developing new molecular MRI contrast agents using CION as the platform material.

## Supplementary Material

1

2

## Figures and Tables

**Fig. 8.**
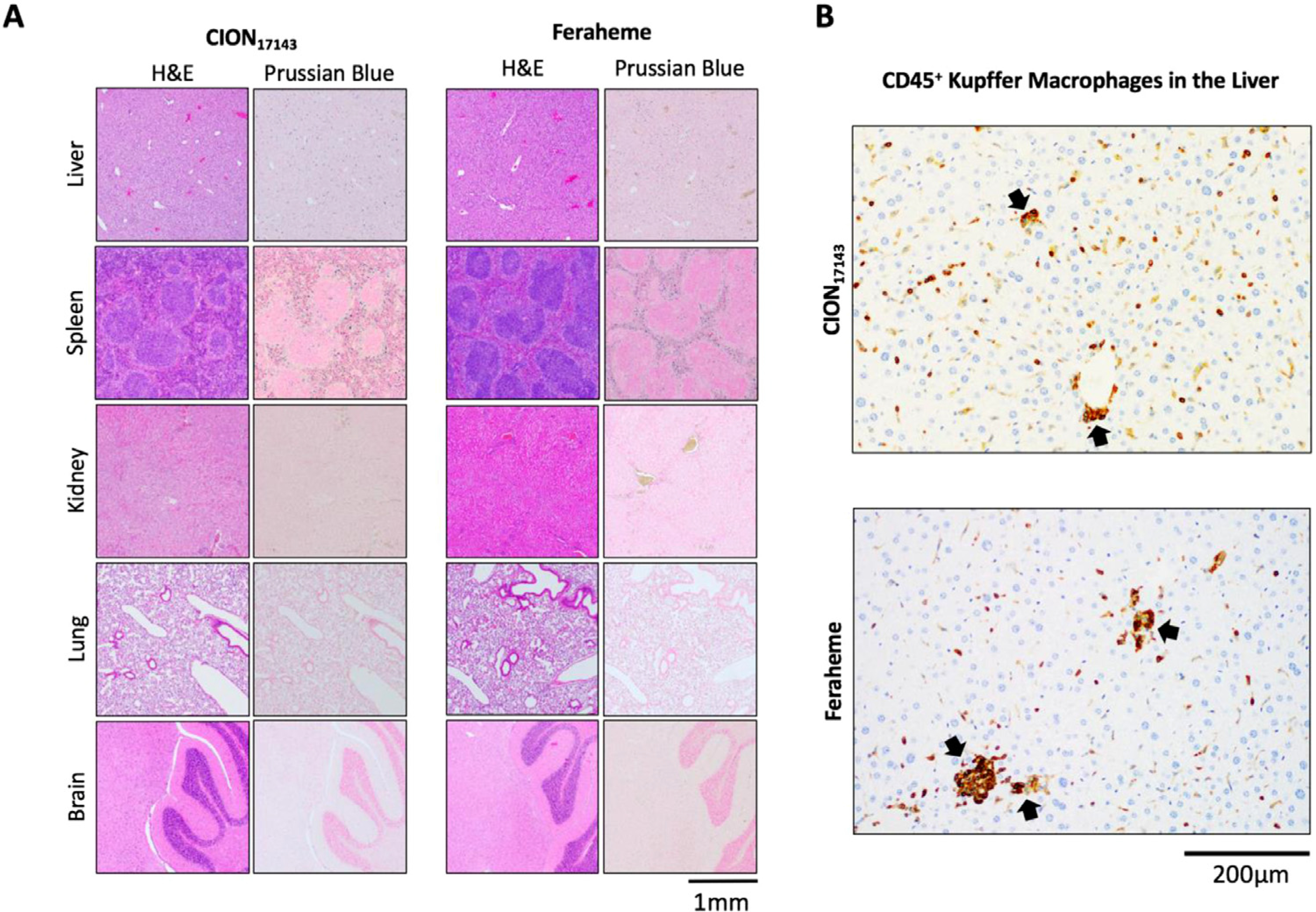
Toxicity and organ deposition of CION_17143_ (*n* = 5) and Feraheme (*n* = 5) in mice 7 days after administration of a bolus 40 mg/kg dose. **(A)** Histological staining with H&E and Prussian Blue were conducted on sectioned liver, spleen, kidney, lung and brain showing no gross organ toxicity. Iron-deposition was restricted to cells in the liver and spleen. **(B)** Immunohistochemistry against CD45^+^ Kupffer macrophages in the liver revealed both CION_17143_ and Feraheme displayed comparable mild lymphocytic inflammation (black arrows).

## Data Availability

The raw and processed data required to reproduce these findings are available to download from https://data.mendeley.com/datasets/6mgrb9ttkn/2. CION samples may be available free-of-charge to the reader of this article for research purpose until supplies last. For more information, please visit https://camri.org or contact camri@unc.edu.
