# Supplementary material for: One-pot synthesis of carboxymethyl-dextran coated iron oxide nanoparticles (CION) for preclinical fMRI and MRA applications
Source: Neuroimage. Author manuscript; Available in PMC 2021 Sep 4. (PMC8418149; doi:10.1016/j.neuroimage.2021.118213)
Supplement: 1 [file NIHMS1730645-supplement-1.docx]

**Supplementary Materials**

**One-Pot Synthesis of Carboxymethyl-Dextran Coated Iron Oxide Nanoparticles (CION) for Preclinical fMRI and MRA Applications**

Manasmita Das^1-3^*, Esteban A. Oyarzabal^1-4^*, Lars Chen^1-3^, Sung-Ho Lee^1-3^, Neal Shah^1-3^, Gabby Gerlach^1-3^, Weiting Zhang^1-3^, Tzu-Hao Harry Chao^1-3^, Nathalie Van Den Berg^1-3,7^, Carolyn Liu^1-3^, Carrie Donley^5^, Stephanie A. Montgomery^6^, and Yen-Yu Ian Shih^1-3^

^1^Center for Animal MRI, University of North Carolina, Chapel Hill, NC, USA

^2^Biomedical Research Imaging Center, University of North Carolina, Chapel Hill, NC, USA

^3^Department of Neurology, University of North Carolina, Chapel Hill, NC, USA

^4^Curriculum in Neurobiology, University of North Carolina, Chapel Hill, NC, USA

^5^Center for Nanotechnology in Drug Delivery, University of North Carolina, Chapel Hill, NC, USA

^6^Department of Pathology and Laboratory Medicine, University of North Carolina, Chapel Hill, NC, USA

^7^Medical Image and Signal Processing Group, Ghent University, Ghent, Belgium

**Supplemental Methods**

**Fluorescent labeling of CION with Rhodamine B.** To label CION with Rhodamine B, 200 mg (0.418 mmol) of Rhodamine B was dissolved in minimum volume of acetonitrile in a 50 ml beaker and activated with 1-ethyl-3-(3-(dimethylamino) propyl) carbodiimidehydrochloride (EDC· HCl, 104 mg, 0.543 mmol) in the presence of bicarbonate coupling buffer (0.5 ml). After stirring the reaction mixture for 15 h in darkness, 0.3 ml 2,2′-(ethylenedioxy)-bis-(ethylamine) (EDEA) was added to the reaction mixture and left to stirring at 25°C for another 24 h in darkness. In a separate beaker, a colloidal suspension of CION_17143_ in PBS (4 ml, 17 mg Fe/ml) was activated with 25 mg of EDC in presence of bicarbonate coupling buffer. After 24 h of continuous stirring, EDC activated CION_17143_ was added to the contents of the first beaker and stirred for another 24h. Thereafter, the reaction mixture was poured to a 30 ml Slide-A-Lyzer dialysis cassette and dialyzed against PBS for 4h to remove the unreacted dye and other reagents as well biproducts from the nanoparticles. The purified, fluorescent-tagged CION_17143_ was freeze-dried and stored at 4°C for further studies.

**Table S1**. List of Equipment, Laboratory Supplies and Reagents required for CION synthesis

1. List of Equipment and Labwares required to establish a basic CION synthesis environment

| S/No | Equipment  (One-Time Purchase) | Source * | Price (USD) | Number required | Total Cost  (USD) |
| --- | --- | --- | --- | --- | --- |
| 1. | Fisher Scientific™ Isotemp™ Stirring Hotplates | Fischer Scientific | 539 | 1 | 539 |
| Accessories | | | | | |
| a. | Fisherbrand™ Transparent Shield | Fischer Scientific | 23.35 | 2 | 46.7 |
| b. | Support Rod  (12mm dia., 400mm, M10) | Fischer Scientific | 39.90 | 2 | 79.8 |
| c. | C-5, Clamp Holder  (PP body, 12 mm dia.) | Fischer Scientific | 8.86 | 2 | 17.72 |
| d. | 3 Prong Clamp  (60 mm Grip) | Fischer Scientific | 15.51 | 2 | 31.02 |
| e. | Fisherbrand™ Octagon Spingarn™ Magnetic Stirring Bars | Fischer Scientific | 8.81 | 2 | 17.62 |
| f. | Fisherbrand™ Magnetic Stir Bar (Six Packs) | Fischer Scientific | 36.40 /  6 Pack | 1 | 36.40 |
| 3. | [Caframo BDC250 Petite Digital Overhead Stirrers with 2 Impellers, 1/100 HP, 2 L, 100-240V.](https://www.amazon.com/gp/product/B00UW57ZB0/ref=ox_sc_act_title_5?ie=UTF8&psc=1&smid=A1GJ9URVN5UPZI) | Thomas Scientific | 392.34 | 1 | 392.34 |
| 4. | Branson CPX series ultrasonic bath with digital timer, bath volume 1.9 L | Millipore Sigma | 556.20 | 1 | 556.20 |
| 5. | UNICO Powerspin LX Centrifuge, Variable Speed 300-4,000 RPM, 6 Place, 30 Min. Timer, 6X10Ml Or 3X15Ml Capacity with 18 Place Tube Holdster Rack C856H | Amazon | 492.66 | 1 | 492.66 |
| 6. | Fisherbrand™ Mini Vortex Mixer | Fischer Scientific | 252.88 | 1 | 252.88 |
| 7. | [200 / 0.0001g 0.1mg Digital Analytical Balance Weighing Precision Lab Scale 110V](https://www.amazon.com/0-0001g-Digital-Analytical-Weighing-Precision/dp/B01GR85IH8/ref=sr_1_12?keywords=weighing+balance&qid=1574101904&s=industrial&sr=1-12) | Amazon | 430 | 1 | 430 |
| Labware (One-Time Purchase) | | | | | |
|  | Borosilicate Glass Beaker Set (Pack of 6) - Graduated Low Form Measuring Beakers in Various Sizes (25/50/100/250/500/1000 ml) | Amazon | 19.95 | 2 | 39.9 |
|  | SUPERLELE Thick Glass Beakers 500ml, 1000ml, 2000ml, Borosilicate Glass Graduated Measuring Beaker Set, Large Capacity Low Form Griffin Thick Wall Type Beakers with brush | Amazon | 22.99 | 1 | 22.99 |
|  | Fisherbrand™ General Purpose Liquid-in-Glass Partial Immersion Thermometers | Fischer Scientific | 12.25 | 2 | 24.5 |
|  | 12Pcs Stainless Steel Lab Spatula Micro Scoop Reagent Laboratory Mixing Spatula 22cm Long Sampling Spoon | Amazon | 11.99 | 1 | 11.99 |
|  |  |  |  | **Total cost** | **$2,998. 72**** |

*The source of the equipment and labware as well as their specifications are suggestive only. The information here provides a general idea for those intend to set up their own CION synthesis environment. This should not be used as a definitive budget estimate.

** The total cost estimated here is tentative. Price may vary depending on geographical location, vendors, specification of the instrument procured etc.

1. List of Laboratory Supplies and Reagents required to synthesize CION in-house

**---------------------------------------------------------------------------------------------------------------------**

**CALCULATIONS:**

4 mmol of Fe (III) salt is reacted with 2 mmol of Fe (II) salt in presence of 3.5 g of carboxymethyl-dextran sodium salt to produce CION_17143_ containing 6 mmol of Fe.

- Because some iron will be wasted during the synthesis and purification steps, we assume that 4 mmol of Fe(III) and 2 mmol of Fe (II) salts react in presence of CMD to produce CION_17143_ comprised of approximately 300 mg of Fe (theoretically).
- Therefore, in order to produce 150 ml of CION_17143_ with a concentration of 20 mg Fe/ml, 3000 mg Fe would be the target, equivalent to 10 times scale up of 300 mg of Fe

Amount of Fe (III) salt (FeCl_3_. 6 H_2_O, MW: 270.30) required: 40 mmol = 40* 270.30 = 10. 812 g.

Amount of Fe (II) salt ( FeSO_4_. (NH_4_)_2_SO_4_. 6 H_2_O, MW: 392.14) required: 20 mmol = 2042 * 392.14 = 7842. 8 = 7.842 g.

Amount of CMD salt required to synthesize CION_17143,_ MW: 10 – 20 kDa: 35 g.

The tentative cost for synthesizing 150 ml of CION_17143_ with a concentration of 20 mg Fe/ml is estimated accordingly.

To synthesize other CION variants, namely, CION_60000_ or CION_30000_ , 10 g and 20 g of CMD will be required, which reduces the overall cost of synthesis.

---------------------------------------------------------------------------------------------------------------------

| S/No | Laboratory Supplies/ Reagents | Source | Price (USD)/ Quantity | Amount required | Total cost in USD |
| --- | --- | --- | --- | --- | --- |
| Reagents | | | | | |
| 1. | Iron (III) chloride hexahydrate (FeCl_3_.6H_2_O) | Millipore Sigma (Sigma Aldrich) | 108.00/500 g | 10.812 | 2.34 |
| 2. | Ammonium iron (II) sulfate hexahydrate [FeSO_4_. (NH_4_)_2_SO_4_. 6 H_2_O] | Millipore Sigma | 38.90/100 g | 7.842 | 3.05 |
| 3. | Carboxymethyl-dextran (CMD) Sodium Salt | Millipore Sigma/ MILWAUKEE | 492.4/ 100 g | 35 | 172.34 |
| 4. | Ammonium Hydroxide Solution | Millipore Sigma | 39.40/ 500 g | 20 ml | 1.576 |
| 5. | Phosphate buffered saline tablets | Fischer Scientific/ Research Products International Corp | 49.20/ 100 tablets | 5 Pcs or synthesis + dialysis | 2.46 |
| 6. | Hydrochloric acid (for occasional pH adjustment and removing iron stains from glass apparatus) | Millipore Sigma | 69.10/500 ml | 10 ml | 1.382 |
| Laboratory Supplies | | | | | |
| 1. | Slide-A-Liner™ 20K MWCO G2 Dialysis Cassettes, 70 ml capacity | Fischer Thermo Scientific | 199.00 / pack of 6 | 3 | 99.5 |
| 2. | GE Healthcare Whatman™ Puradisc 25mm PTFE Syringe Filters | Fischer Thermo Scientific | 334.00/  pack of 50 | 2 | 13.36 |
| 3. | 10ml Sterility Tested Injection Clear Glass Vials with Red Aluminum Seals | Amazon | 25.99/ pack of 25 | 10 | 10.396 |
| 4. | 2.5ml/cc Disposable Sterile Syringe with 23Ga Needle, Single Aseptic and Separate Packaging | Amazon | 9.80/ 20 Pack | 2 | 0.98 |
| 5. | 18G/1.5In/38mm (18G-100Pack) | Amazon | 13.99 | 2 | 0.2798 |
| 6. | 15 ml Falcon Centrifuge Tubes | Amazon | 39/ 50 pcs |  |  |
| 7. | GE Healthcare Whatman™ pH Indicator Papers, CF Strips (pH range 0-14) | Fischer Scientific | 39.80/ 100 Pcs | 1 | 0.398 |
| 8. | Polypropylene 1.5 ml Micro-centrifuge Tube with Snap Cap, Natural (Pack of 500) | Amazon | 12.95/500 Pcs | 10 | 0.259 |
|  | Electricity cost |  |  |  | 20 |
|  |  |  |  | **Total cost** | **$328.32** |

Based on the calculations above, the tentative cost of all equipment and labware needed to perform in-house CION synthesis is ~ $3,000.00 USD and the cost of supplies/reagents needed to synthesize a batch of 150 ml of 20 mg Fe/ml CION _17143_ (i.e. 3000 mg CION _17143_) is ~ $330.00 USD. At the time of this manuscript submission, Feraheme is distributed in 17 ml vials concentrated at 30 mg Fe/ml and costs $400.00 USD per vial at a subsidized cost from UNC hospital pharmacy.

**Table S2.**

**A.** Hydrodynamics size and PDI of CION synthesized with different molar stoichiometric ratio of iron precursors to CMD

| Formulation | Fe (mmol) | CMD (g) | [Fe]:[CMD] | DLS (nm) | PDI |
| --- | --- | --- | --- | --- | --- |
| CION_120000:1_ | 6 | 0.5 | 120000:1 | agglomerate | NA |
| CION_60000:1_ | 6 | 1 | 60000:1 | 365.36 ±5.04 | 0.423±0.021 |
| CION_30000:1_ | 6 | 2 | 30000:1 | 103.47±2.05 | 0.188±0.005 |
| CION_17143:1_ | 6 | 3.5 | 17143:1 | 50.65 ± 2.50 | 0.240±0.018 |
| CION_12000:1_ | 6 | 5 | 12000:1 | 96.30 ± 2.67 | 0.192±0.0123 |

1. Comparison of hydrodynamic diameters of various CION formulations

| **Tukey's multiple comparisons test** | **Mean Diff.** | **95.00% CI of diff.** | **Significant?** | **Summary** | **Adjusted P Value** |
| --- | --- | --- | --- | --- | --- |
| CION_60000_ vs. CION_30000_ | 261.9 | 242.5 to 281.3 | Yes | **** | <0.0001 |
| CION_60000_ vs. CION_17143_ | 314.7 | 297.0 to 332.4 | Yes | **** | <0.0001 |
| CION_60000_ vs. CION_12000_ | 269.1 | 250.7 to 287.4 | Yes | **** | <0.0001 |
| CION_30000_ vs. CION_17143_ | 52.82 | 39.08 to 66.56 | Yes | *** | <0.0001 |
| CION_30000_ vs. CION_12000_ | 7.169 | -7.313 to 21.65 | No | ns | 0.3934 |
| CION_17143_ vs. CION_12000_ | -45.65 | -57.77 to -33.54 | Yes | *** | <0.0001 |

**C**. Comparison of PDI of various CION formulation

| **Tukey's multiple comparisons test** | **Mean Diff.** | **95.00% CI of diff.** | **Significant?** | **Summary** | **Adjusted P Value** |
| --- | --- | --- | --- | --- | --- |
| CION_60000_ vs. CION_30000_ | 0.2351 | 0.1078 to 0.3624 | Yes | ** | 0.0028 |
| CION_60000_ vs. CION_17143_ | 0.1824 | 0.06618 to 0.2986 | Yes | ** | 0.0065 |
| CION_60000_ vs. CION_12000_ | 0.2301 | 0.1101 to 0.3502 | Yes | ** | 0.0023 |
| CION_30000_ vs. CION_17143_ | -0.05270 | -0.1427 to 0.03732 | No | ns | 0.2763 |
| CION_30000_ vs. CION_12000_ | -0.004967 | -0.09986 to 0.08993 | No | ns | 0.9977 |
| CION_17143_ vs. CION_12000_ | 0.04773 | -0.03166 to 0.1271 | No | ns | 0.2592 |

**Table S3.** Surface elemental composition of CION prepared with various molar stoichiometric ratio of Fe: CMD

| Formulation | [CMD (mmol)]:[Fe(mmol)] | C:Fe (atomic concentration %) |
| --- | --- | --- |
| CION_60000_ | 1.66 × 10^-5^ | 96.21565 ± 7.646419 |
| CION_17143_ | 5.83× 10^-5^ | 134.3611± 1.361111 |
| CION_12000_ | 8.333× 10^-5^ | 436.4688±85.53125 |

**Supplementary Figures**

**
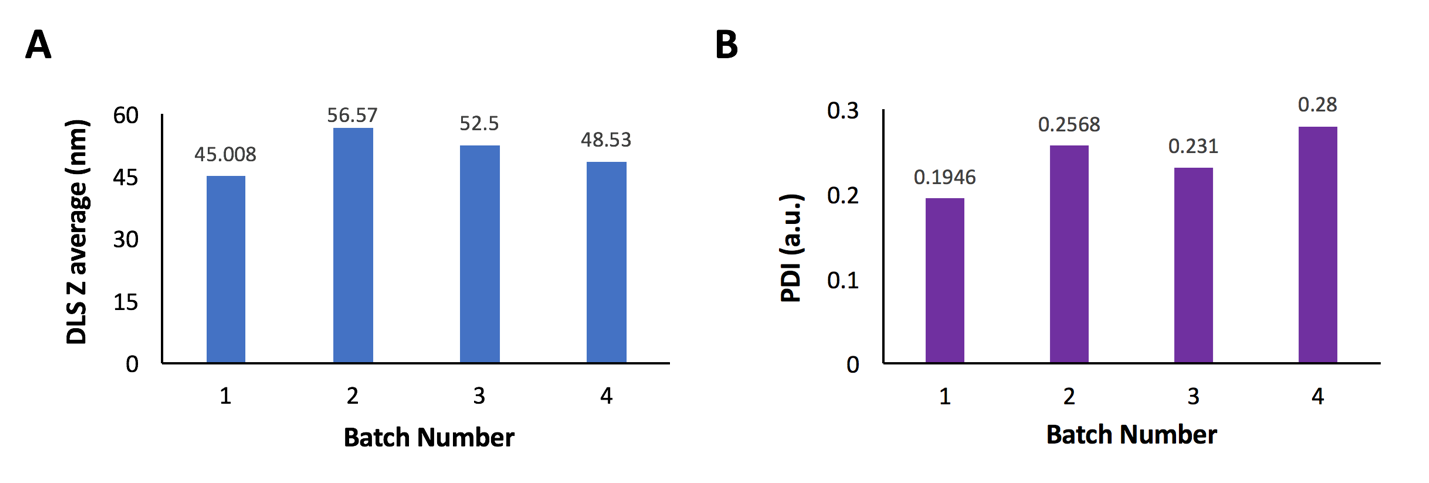
**

**Figure S1.** CION_17143_ synthesis tested from four different batches produced nanoparticles with similar (**A**) dynamic light scattering Z-averages (nm) and (**B**) polydispersity index.


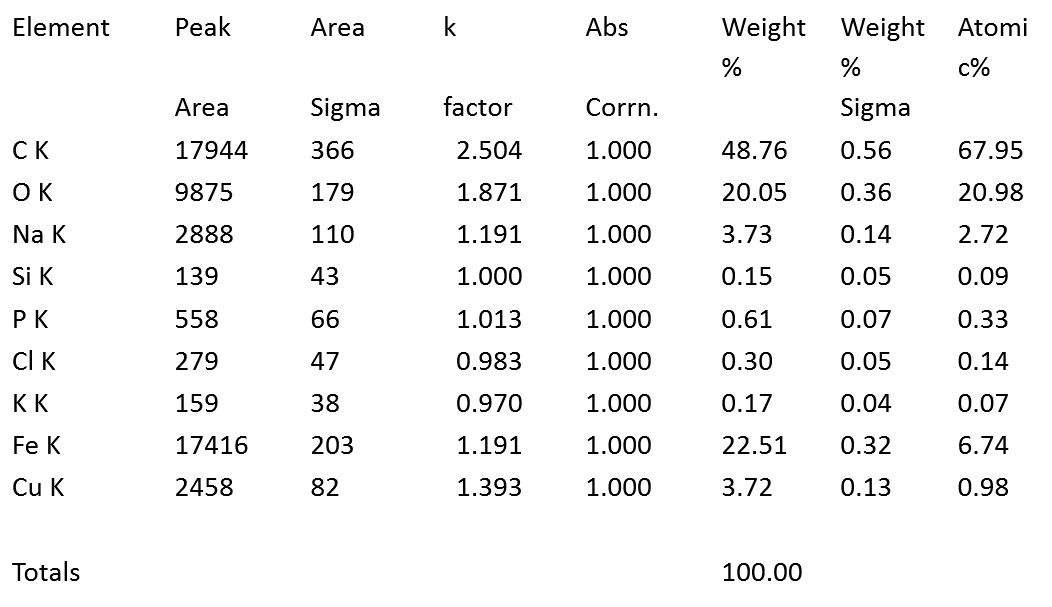

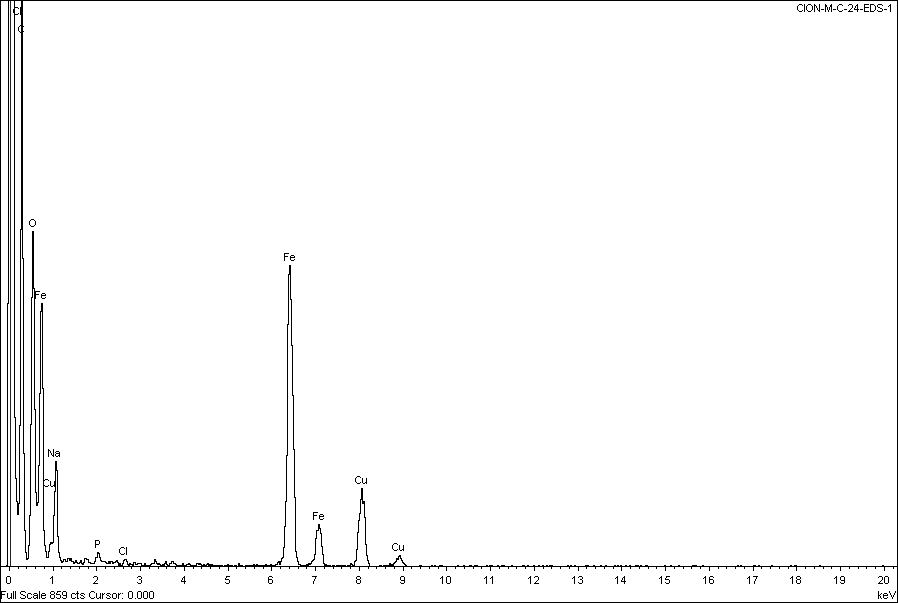


**Figure S2.** Representative electron-dispersive X-ray spectrum of CION_17143_.

**Figure S3.** Surface elemental composition (C: Fe molar stoichiometric ratio) of different CION formulations.

**
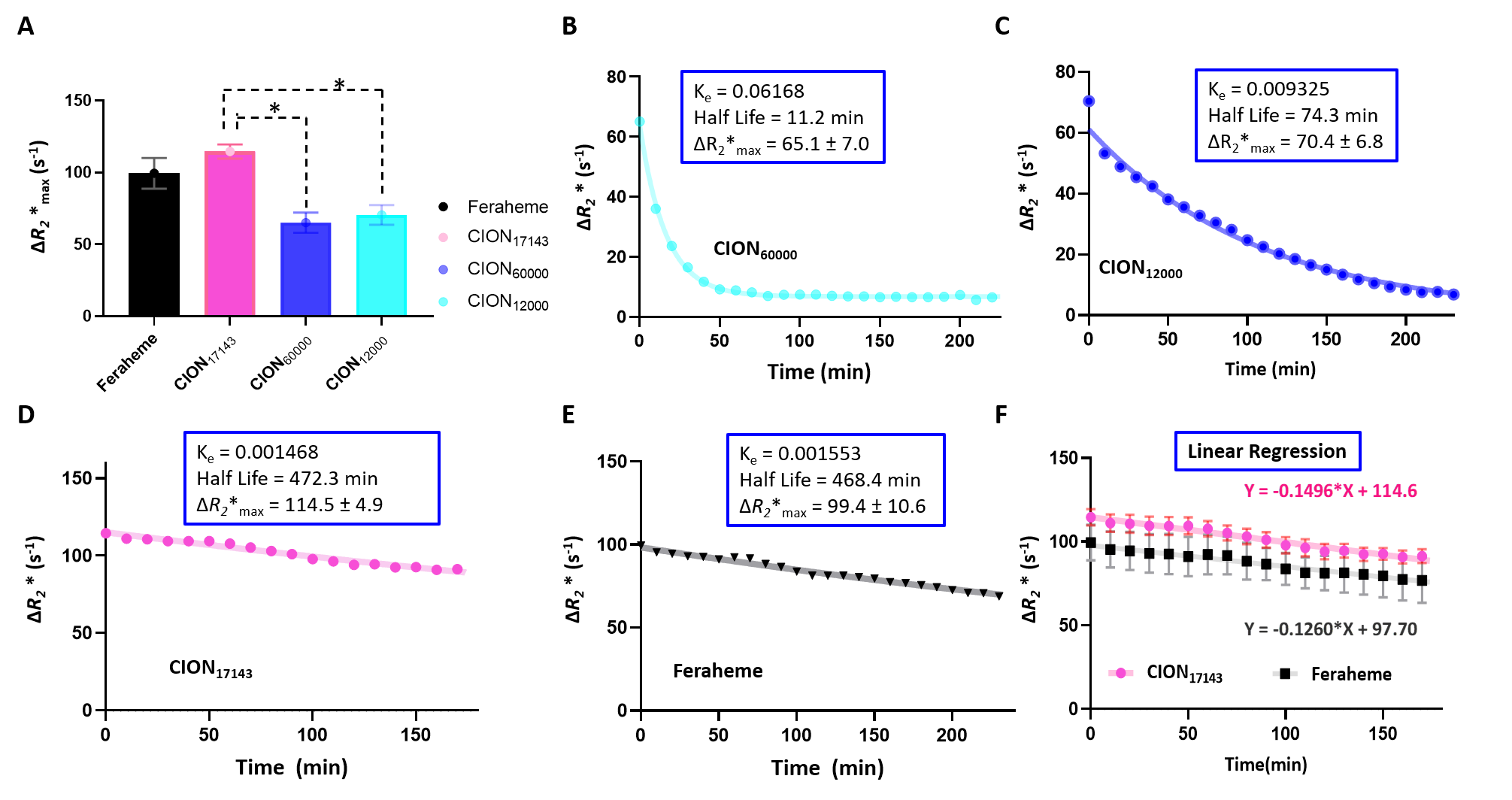
**

**Figure S4. Pharmacokinetic profile of CION in hippocampus**. **(A)** Comparison of Δ*R_2_**_max_ of various CION formulations. **(B-E)** Exponential curve fitting of hippocampal Δ*R_2_** signal changes as function of time in rats treated with CION _60000,_ CION _12000_, CION _17143_ and Feraheme respectively. **(F)** Linear fit of hippocampal Δ*R_2_** signal changes as function of time in rats treated with CION _17143_ and Feraheme.

**
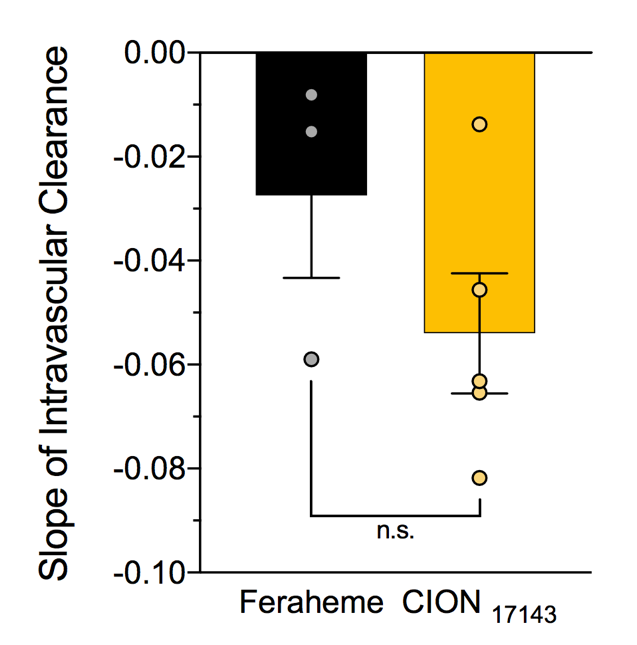
**

**Figure S5.** Slope of intravascular clearance comparison between Feraheme and CION_17143_ in hippocampus.


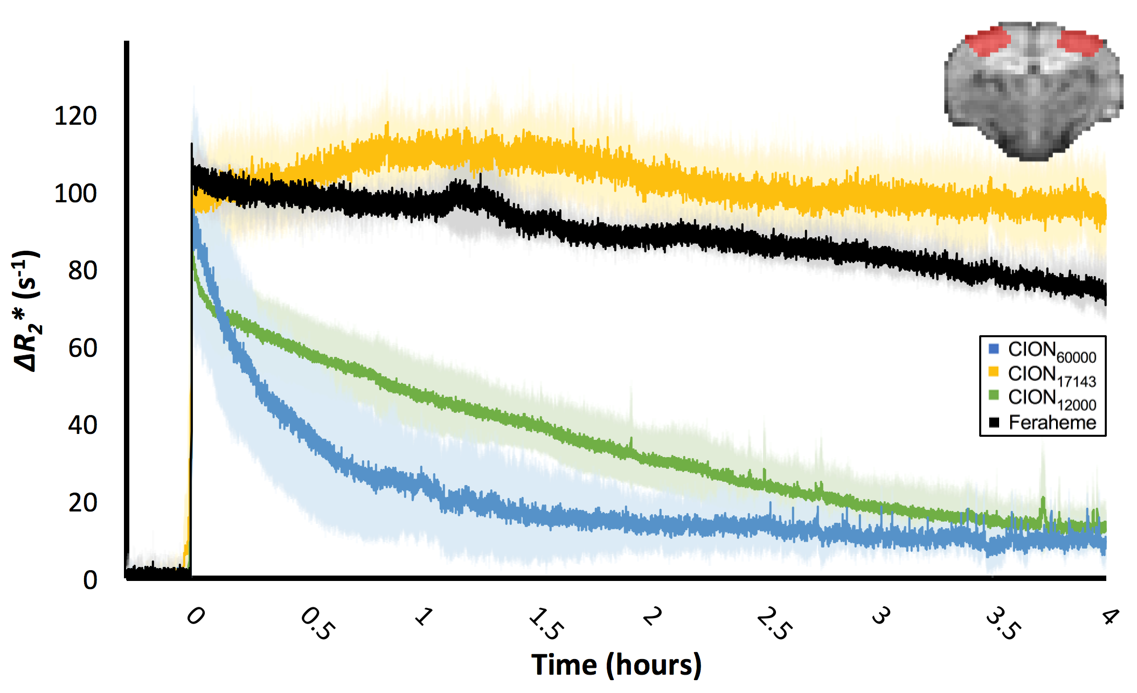


**Figure S6.** Pharmacokinetic profile of CION formulations and Feraheme in the primary somatosensory cortex.

**
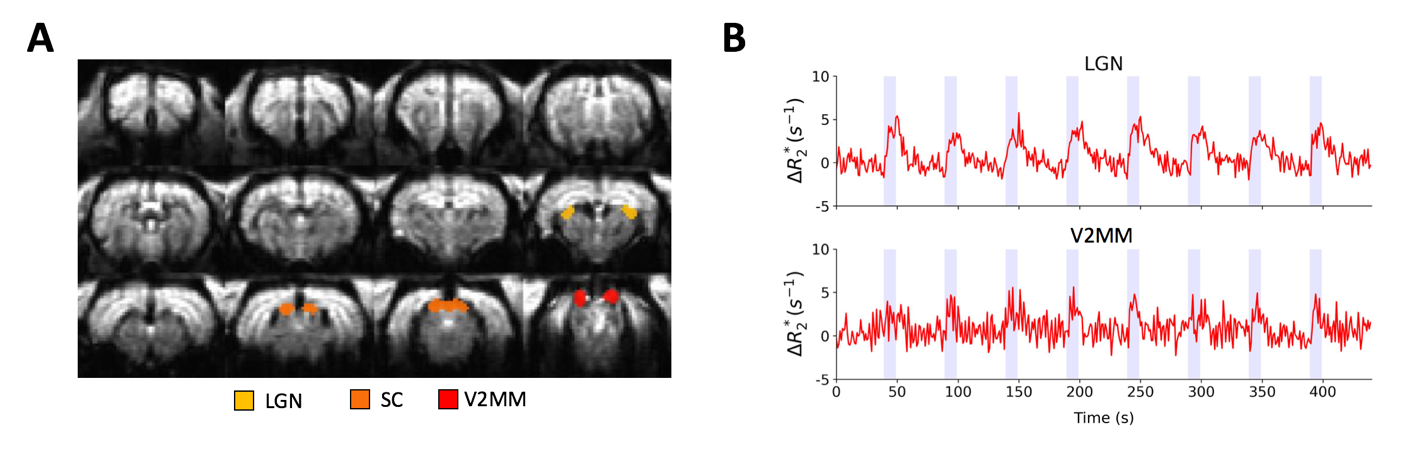
**

**Figure S7.** Additional brain regions showing fMRI responses to visual stimulation. (**A**) ROIs of visual network brain regions, including the lateral geniculate nucleus (LGN), superior colliculus (SC) and mediomedial secondary visual cortex (V2MM). (**B**) Time-locked ∆R_2_* changes in response to visual stimulation epochs. SC responses is shown in Figure 5B of the main manuscript.

**Figure S8.** Body-weight of mouse after injection of CION_17143_
